# Distinctive Profile of IsomiR Expression and Novel MicroRNAs in Rat Heart Left Ventricle

**DOI:** 10.1371/journal.pone.0065809

**Published:** 2013-06-14

**Authors:** Mary K. McGahon, Janet M. Yarham, Aideen Daly, Jasenka Guduric-Fuchs, Lyndsey J. Ferguson, David A. Simpson, Anthony Collins

**Affiliations:** Centre for Vision and Vascular Science, Queen’s University Belfast, Belfast, County Antrim, United Kingdom; IRCCS-Policlinico San Donato, Italy

## Abstract

MicroRNAs (miRNAs) are single-stranded non-coding RNAs that negatively regulate target gene expression through mRNA cleavage or translational repression. There is mounting evidence that they play critical roles in heart disease. The expression of known miRNAs in the heart has been studied at length by microarray and quantitative PCR but it is becoming evident that microRNA isoforms (isomiRs) are potentially physiologically important. It is well known that left ventricular (patho)physiology is influenced by transmural heterogeneity of cardiomyocyte phenotype, and this likely reflects underlying heterogeneity of gene expression. Given the significant role of miRNAs in regulating gene expression, knowledge of how the miRNA profile varies across the ventricular wall will be crucial to better understand the mechanisms governing transmural physiological heterogeneity. To determinine miRNA/isomiR expression profiles in the rat heart we investigated tissue from different locations across the left ventricular wall using deep sequencing. We detected significant quantities of 145 known rat miRNAs and 68 potential novel orthologs of known miRNAs, in mature, mature* and isomiR formation. Many isomiRs were detected at a higher frequency than their canonical sequence in miRBase and have different predicted targets. The most common miR-133a isomiR was more effective at targeting a construct containing a sequence from the gelsolin gene than was canonical miR-133a, as determined by dual-fluorescence assay. We identified a novel rat miR-1 homolog from a second miR-1 gene; and a novel rat miRNA similar to miR-676. We also cloned and sequenced the rat miR-486 gene which is not in miRBase (v18). Signalling pathways predicted to be targeted by the most highly detected miRNAs include Ubiquitin-mediated Proteolysis, Mitogen-Activated Protein Kinase, Regulation of Actin Cytoskeleton, Wnt signalling, Calcium Signalling, Gap junctions and Arrhythmogenic Right Ventricular Cardiomyopathy. Most miRNAs are not expressed in a gradient across the ventricular wall, with exceptions including miR-10b, miR-21, miR-99b and miR-486.

## Introduction

MicroRNAs (miRNAs) are single-stranded non-coding RNAs consisting of 18–25 nucleotides (nts) that negatively regulate target gene expression through mRNA cleavage or translational repression [1]. In recent years miRNAs have been implicated in the control of expression of many proteins in development and pathology. In particular there is mounting evidence that they play critical roles in diseases of the heart [2]–[4], and they have great potential as circulating biomarkers of cardiac damage [5].

The rat heart has provided a useful model for the study of heart physiology and disease for many years, and this utility has been extended to investigations into the role of miRNAs. Numerous recent studies have used the rat heart and rat heart cells to demonstrate the involvement of miRNAs in ischaemia [6]–[20], hypertrophy [21]–[29], heart failure [30]–[34], fibrosis [35]–[38], apoptosis [6], [10], [17], [39]–[43], metabolism [44], [45], angiogenesis [11], [46]–[48], drug action [11], [15], [16], [49]–[51] and arrhythmogenesis [35], [51].

Analysis of sequencing-based miRNA expression data has revealed that individual miRNAs exist not just as a single canonical sequence but exhibit a range of sequence variants or ‘isomiRs’ [52]–[55]. These isomiRs can have additions or truncations at their 5′ or 3′ end, or internal single-base differences. The variation between isomiRs occurs during biogenesis due to cleavage by the RNaseIII enzymes Dicer and/or Drosha at alternative sites or post-transcriptional editing. The availability of the rat whole genome sequence makes it possible to differentiate isomiRs of a single microRNA from closely related microRNAs with very similar sequences that originate from separate miRNA genes, designated ‘a’, ‘b’, etc. (for some microRNAs several genes exist which result in the same mature sequence and in this case it is not possible to determine whether specific isomiRs are formed preferentially from one gene). The distribution of isomiRs indicates that they are not randomly generated, and may have (patho)physiological importance [56]–[58]. Indeed, specific isomiRs have been shown to be functional and to have target gene repertoires that differ from those of their corresponding canonical miRNAs [59], [60]. Recent data suggests the possibility of isomiR-specific gene targeting in the heart [61], [62].

The vast majority of myocardial miRNA expression profiling studies to date have used microarray technologies or quantitative PCR (qPCR). Although much useful data has emerged from these studies, these techniques, like all methodological approaches, have limitations. Microarrays and qPCR assays are designed to detect and quantify molecular species that are already known to exist; novel sequences are not detected. IsomiRs would either be indistinguishable from the canonical sequence or not detected at all. The former is likely in microarray-based methods while the latter could apply in qPCR, depending on the type of isomiR. In contrast to the aforementioned methods ‘next-generation’ or ‘deep’ sequencing is able to detect and quantify novel sequence variants as well as those already known. We therefore used next-generation sequencing to characterise the expression profile of miRNAs in rat heart left ventricular wall, identify novel miRNAs and novel orthologs not previously characterised in this species and determine the relative expression of isomiRs of individual miRNAs. To our knowledge this is the first report of miRNA deep sequencing data from rat heart using the Illumina platform. We detected several novel rat miRNAs and multiple novel isomiRs of known miRNAs, many of which were detected at a higher frequency than the corresponding mature sequence recorded in miRBase [63], [64].

The well established heterogeneity of cardiomyocyte physiology across the left ventricular wall influences both normal heart function and pathophysiology [65], [66] and likely reflects underlying heterogeneous gene expression. Given the significant role of miRNAs in regulating gene expression, knowledge of how the miRNA profile varies across the ventricular wall will be crucial to understand the mechanisms governing transmural physiological heterogeneity. Data describing transmural miRNA expression is very limited and we therefore undertook the first comprehensive determination of miRNA expression profiles at different locations across the left ventricular wall. We have found that most miRNAs are not expressed in a gradient across the ventricular wall, with the exceptions of miR-10b, miR-21, miR-99b and miR-486.

## Methods

### Ethics Statement

This study was carried out in strict accordance with the recommendations in the Guide for the Care and Use of Laboratory Animals of the National Institutes of Health; and the United Kingdom Animals (Scientific Procedures) Act, 1986. The protocol was approved by the Animal Research Ethics Committee of Queen’s University Belfast (Project Licence: PPL 2683).

### miRNA Library Preparation

The hearts of 3 male 8 month old Sprague-Dawley rats were rapidly extracted after euthanasia with sodium pentobarbital. A section of the free wall of the left ventricle was dissected into epicardium, mid-myocardium and endocardium by cutting approximately 1 mm from the epicardial and endocardial surfaces. Tissue was weighed, chopped, stored in RNAlater RNA stabilisation reagent (Qiagen, Crawley UK) and then flash frozen in liquid nitrogen before placing in storage at −80°C. Small RNA was extracted (miRNeasy Kit; Qiagen), quantified (Nanodrop; Thermo Scientific) and quality assessed for degradation (RNA Nano Chip, Bioanalyser 2100; Aligent Technologies, Wokingham UK; only samples with a RNA integrity no. (RIN) ≥8 were carried forward) and retention of small RNA (Small RNA Chip, Bioanalyser 2100). Small RNA was preferentially ligated with adapters, reverse transcribed into cDNA and amplified with 9 individually tagged primer indices (TruSeq Small RNA Sample Preparation Kit; Illumina, Little Chesterford, UK) and a library of small RNA created for each sample. After gel purification the cDNA products were again analysed on the bioanalyser using a High Sensitivity DNA Chip and assessed for the presence and concentration of the peak corresponding to ligated and tagged miRNA (approximately 147 nt). It was observed that the successful presence of an appropriate end product was not wholly dependent on the integrity of the initial RNA sample (RIN; See Fig. S1) but could be better predicted from the presence or relative absence of a range of small RNA species (tRNA, 5S and miRNA) assessed on the Small RNA Chip electropherogram; therefore only samples with suitable RIN values exhibiting good retention of small RNA species were used for library preparation. After pooling, the samples were sequenced by TrinSeq (Trinity Genome Sequencing Lab & Neuropsychiatric Genetics Group, Trinity College Dublin, Ireland (http://www.medicine.tcd.ie/sequencing); using TruSeq SR Cluster Kit v5 (Illumina) and the resultant data trimmed and aligned to miRBase v18 (CLC Genomics Workbench v4.0; CLC bio, Swansea UK).

### PCR

Genomic DNA was extracted from rat heart (Direct PCR lysis reagent; Viagen Biotech Inc, Los Angeles, CA, USA) to form the template for confirmation of the stem loop sequence for pre-miR-486 in a PCR reaction using primers (Table S1) designed from a rat genomic DNA sequence (gnl|ti|1621823597; gnl|ti|1718641223) not present in the most recent build of the rat genome (Baylor 3.4), and DreamTaq polymerase (Fermentas). Cycling parameters were: 95°C for 3 min, 35× (95°C for 30 s, 49°C for 30 s, 72°C for 20 s), 72°C for 15 min. Amplified products were sequenced (Genomics Core, Queen’s University Belfast).

Quantitative PCR was carried out using Taqman small RNA assays and TaqMan gene expression assays (Invitrogen – Life Technologies Ltd, Paisley, UK) on RNA samples from rat hearts dissected and extracted as detailed above. REST 2009 software [67], [68] was used to determine the expression ratio between epicardial and endocardial samples using multiple endogenous controls which were tested for consistent expression across samples using BestKeeper [69].

### Plasmid Construction

A 102 bp fragment of the human gelsolin sequence (NCBI Reference: Chromosome 9, NC_000009.11 (124030380.124095120)) was amplified from human genomic DNA (Bioline, London, UK) by a two-stage nested PCR using Phusion® Hot Start II High-Fidelity DNA Polymerase (see Table S1 for primer sequences).

The product was restricted with *Xho*I and *Bam*HI and ligated into pmR-mCherry (Clontech, Saint-Germain-en-Laye, France; modified as described [70]). The final product (pmChGSN) has the CMV promoter driving transcription of mCherry with the gelsolin fragment as 3′ UTR.

The plasmid pSM30 (encoding EGFP) was used for expression of miRNAs [71]. Complementary pairs of oligonucleotides (25 µM) encoding artificial miRNA sequences (miR-133a canonical isomiR and most common isomiR (miR-133a(v))), a siRNA sequence designed to down regulate mCherry expression (siR-mCh), or a random non-targeting negative control sequence (NTC) were annealed and ligated (T4 DNA ligase; New England Biolabs, Hitchin, UK) into pSM30 restricted with *Bsm*BI (New England Biolabs, Hitchin, UK). Oligonucleotide sequences are shown in Table S2.

Following confirmation of inserts by DNA sequencing (Genomics Core, Queen’s University Belfast, UK), plasmids were prepared with a Maxiprep kit (Qiagen, West Sussex, UK) following the manufacturer’s instructions. Custom oligonucleotides were purchased from Eurogentec (Seraing, Belgium).

### Dual Fluorescence miRNA Targeting Assay

This assay has been described in detail previously [70]. HEK293 cells were plated into a 24-well plate. The following day, cells were transiently transfected with pmChGSN, in addition to one of pSM30miR-133a, pSM30miR-133a(v), pSM30NTC or pSM30siR-mCh using Turbofect Transfection reagent (Thermo Scientific, Hemel Hempstead, UK). Cells were imaged at 20X magnification using a Nikon Eclipse TE2000-U microscope equipped with a 488 nm filter (red; mCherry fluorescence) and a 550 nm filter (green; EGFP fluorescence). Red and green images were acquired of multiple fields using NIS Elements software (Nikon, Badhoevedorp, Netherlands). Cross-channel bleed-through was found to be negligible by acquiring red images of cells transfected with pSM30miR-133a only, and green images of cells transfected with pmChGSN only. These images were then used for subtraction of background fluorescence. Images in tiff format were analysed by Volocity software (Volocity 5.5.1, PerkinElmer Inc., Waltham, MA, USA). We developed a Volocity pipeline for identifying cells in green images and measuring the total red and green intensities of each identified cell. Data were exported in Microsoft Excel format for further analysis (Prism 4, GraphPad, San Diego, CA, USA).

## Results

### Overview of Next Generation Sequencing Data

**Figure 1:**
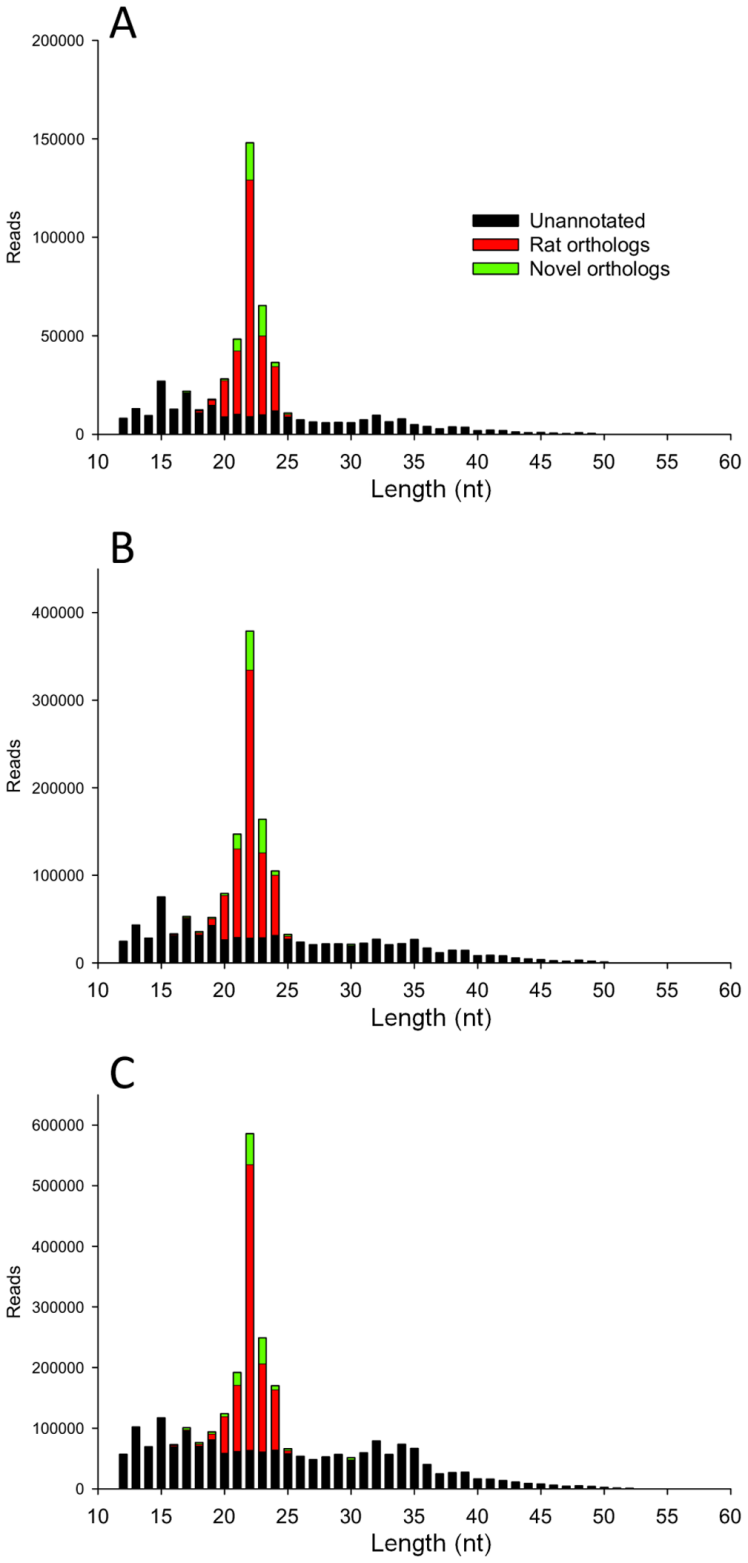
Length distribution analysis of small RNA sequences in 3 mid-myocardial rat samples. Reads greater than 12 nt are included. Red bars represent reads annotated to known rat miRNAs (miRBase v18), green bars represent reads annotated to miRNAs in other species (novel orthologs) and black bars represent unannotated small RNA sequences.

**Figure 2:**
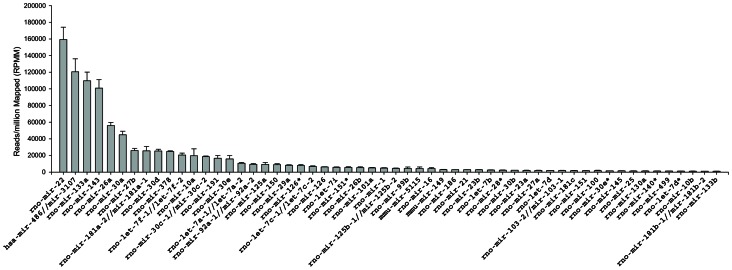
miRNA detection frequency. Most highly detected miRNAs grouped on mature sequence, normalised to total annotated reads (expressed in reads per million mapped (RPMM) from 3 rat mid-myocardial samples).

A total of 2,947,762 reads between 12 and 73 nt in length were produced by next generation sequencing of the 3 samples of mid-myocardium (Fig. 1). miRNAs were aligned and grouped on the mature sequence, allowing for up to 2 mismatches within the sequence and/or 3 additions or deletions from either the 5′ or 3′ ends. This identified 1,782,482 reads that were annotated to known miRNA sequences from *Rattus norvegicus* entries in miRBase v18 and a further 307,672 annotated to miRNA sequences only previously reported in other species (potentially representing novel orthologs). The proportions of reads of length >12 representing known rat miRNAs, potential novel orthologs or not matching any known miRNA (unannotated) are shown in Fig. 1. In all 3 samples the median length of reads was 22 nts and 80.73±0.20% (mean ± sem) of the reads at this length were annotated to known rat miRNA sequences. In total, 145 previously characterised rat miRNA sequences had representation of ≥10 raw reads prior to normalisation (Table S3; S3A compiled from miRNAs with ≥10 raw ‘exact mature’ reads, S3B compiled from miRNAs with <10 ‘exact mature’ reads but ≥10 ‘grouped on mature’ raw reads) in at least one of the 3 samples. Using the same criteria a further 68 potential novel orthologs were identified (Table S4). Additionally all reads of sequences originally reported in miRbase as the minor strand (*) were grouped (upon similar criteria as above; Table S5) resulting in a further 58 miRNAs of interest, 16 of which (including miR-126*, miR-140*, miR-151* and miR-28*) were detected between 1.23 and 99.56 fold higher in the * form than the ‘major’ mature sequence, while miR-501* was not detected in the major mature strand form. By combining the 3 sample groups and normalising to reads per million mapped (RPMM), the most highly detected miRNAs (≥1000 RPMM) were identified (Fig. 2). miR-22, miR-486, miR133a and miR-143 were detected at greater than 100,000 RPMM. Twelve miRNAs were detected at greater than 10,000 RPMM and a further 40 miRNAs were detected at greater than 1,000 RPMM. Six of the top 56 most highly detected miRNAs were * sequences. Fifty-three of the top 56 most highly detected miRNAs were previously annotated rat miRNAs, but the second most highly detected miRNA mapped to the human miRNA hsa-miR-486-5p and has yet to be registered in miRBase as a rat miRNA, although Small *et al.* [72] have shown it to be highly expressed in rat myocardium. Characterisation of the rat miR-486 gene is described below.

Individually, miR-22 has been implicated in the phenylephrine- or angiotensin II-induced hypertrophic reduction in phosphatase and tensin homolog (PTEN) [24]; miR-26a and 30d in calcium dependent cardiac dysfunction [73], and miR-27b in heart development and hypertrophy [74], [75]. PTEN and Foxo1a are known targets of miR-486 [72]. Of particular note in the context of cardiac physiology, miR-486 is predicted by TargetScan 6.2 (Whitehead Institute for Biomedical Research) to target the transcription factor IRX5, which negatively regulates K_v_4.2 K^+^ channel expression [76], [77].

### Abundance of isomiR Sequences

**Figure 3:**
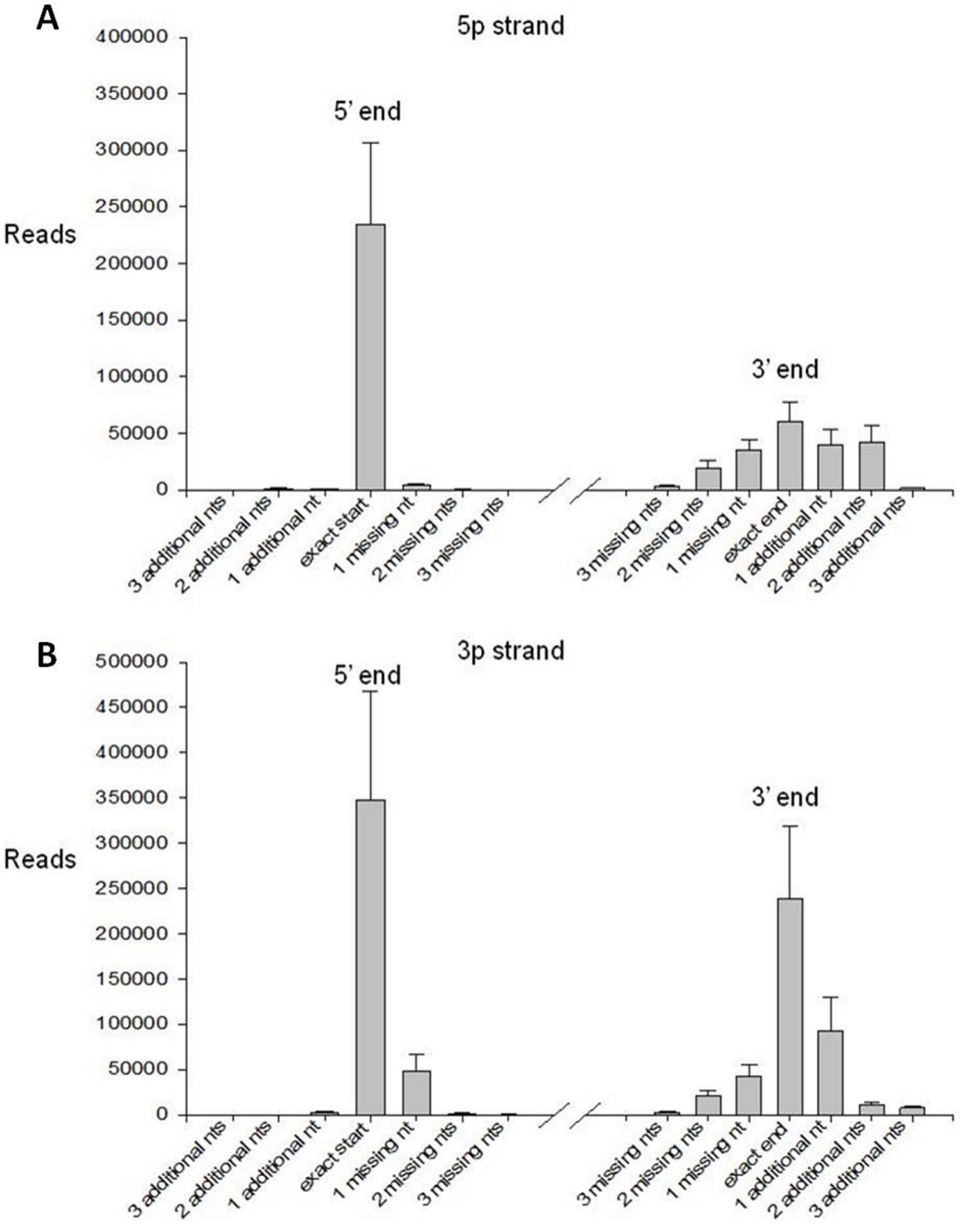
Frequency of isomiRs with 5′ and/or 3′ variations. Distributions of 5′ (left) and 3′ (right) end variants are shown for miRNAs derived from the 5′ arm (A) and from the 3′ arm (B) of the pre-miR hairpin. Data are mean ± SEM for three mid-myocardial samples.

The frequency of isomiRs with 5′ and/or 3′ variations from the canonical (miRBase) sequence is summarised in Fig. 3 for a mid-myocardial sample. Of the sequences derived from the 5′ arm of the hairpin miRNA, 62.6% varied from the canonical sequence at the 3′ end (with approximately half being longer and half being shorter), and 3.6% varied at the 5′ end (with approximately two-thirds being shorter). Of the sequences derived from the 3′ arm, 50.1% varied from the canonical sequence at the 3′ end (with approximately three-fifths being longer), and 13.8% varied at the 5′ end (with a strong bias towards shorter variants). This distribution is similar to that found in cardiomyocyte-derived HL-1 cells [61]. The greater 5′ end variability in the 3' arm-derived compared to 5' arm-derived miRNAs corresponds with the previously reported higher fidelity of Drosha *vs* Dicer cleavage [78]. Variability at the 3' end is also influenced by post-cleavage processing [79], [80].

**Figure 4:**
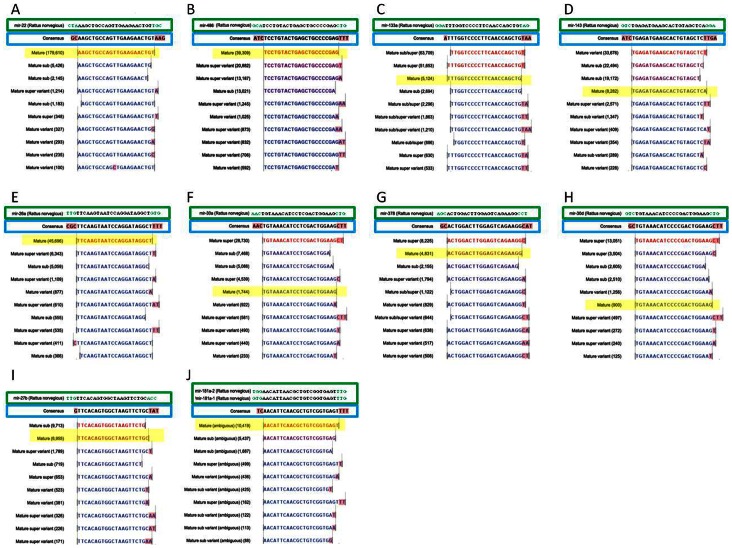
IsomiRs of selected miRNAs. IsomiR sequences of the 10 most highly detected miRNAs (mature miRNA highlighted in yellow) with expression values in brackets from a mid-myocardial sample aligned to the published pre-miR sequence (boxed in green; miRBase v18). Consensus sequence (boxed in blue) for each miRNA represents the most prevalent nt aligned at each position (nts highlighted in pink represent variations from the miRBase published mature sequence).

Closer analysis of the top 56 most highly detected miRNAs revealed that the mature form (or the mature* sequence) of the miRNA as published in miRBase was not always the most common sequence encountered. For the top 10 most highly detected miRNAs, the reads representing the 10 most common isomiR sequences are illustrated in Fig. 4. For only 4 of these, miR-22, 486, 26a and 181a, is the annotated mature form the most highly detected and even for these miRNAs significant numbers of several other isomiRs were also detected. For example, while the exact mature sequence of miR-22 constituted 93.04±0.35% of its total reads, the exact mature sequence of miR-181a only constituted 51.09±0.59%. Characteristically for miRNAs, the majority of miR-486 isomiR sequences differed at the 3′ end of the sequences and were shortened mature sequences (sub) or mature sequences with additional nts (super) matching the pre-miR sequence. However, substantial populations of reads exhibited nt substitutions varying from the pre-miR sequence (compare pre-miR and consensus sequences in Fig. 4B), largely by the addition of nts at the 3′ end, most commonly T’s and A’s.

**Figure 5:**
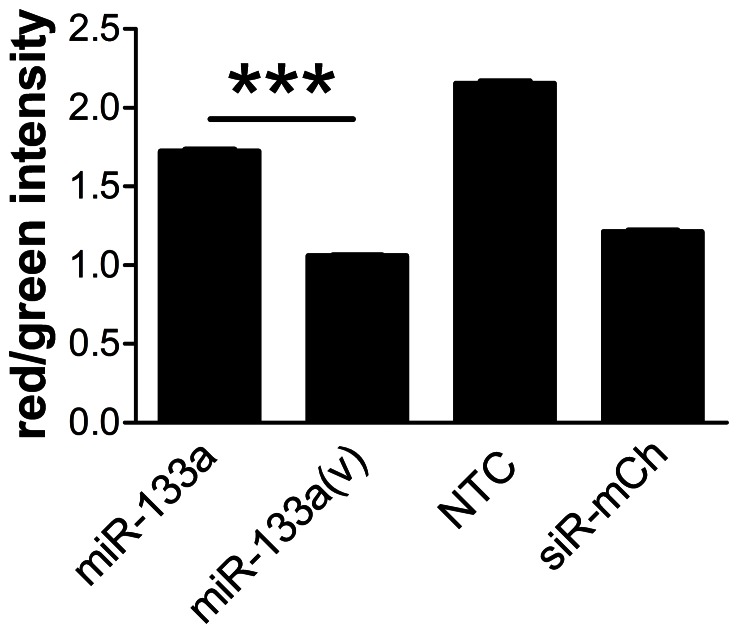
Differential suppression of a gelsolin sequence-tagged reporter gene by miR-133a isomiRs. HEK293 cells were transfected with expression plasmids encoding mCherry with a 3′ partial gelsolin sequence and pSM30 containing inserts for miR-133a, miR-133a(v), a random non-targeting sequence (NTC) or siRNA against mCherry (siR-mCh). Cells were imaged and analysed for both green and red fluorescence intensity. A decrease in red/green ratio *vs* NTC indicates downregulation of target gene expression. Data were log transformed and compared by one-way ANOVA with Bonferroni's Multiple Comparison Test. All pairwise comparisons were significant (p<.001). Comparison of miR-133a *vs* miR-133a(v) is indicated (***). Number of cells (n): miR-133a 4640; miR-133a(v) 2843; NTC 4088; siR-mCh 3884. Representative of 3 separate experiments in which miR-133a(v) was significantly more effective than miR-133a.

Analysis of the sequences attributed to miR-143 (Fig. 4D) showed that the mature sequence was only the 4^th^ most common sequence, indeed the most common miR-143 sequence is an isomiR with an unambiguous post-transcriptional modification, that is a non-templated addition of A at nt no. 22. Additional significantly expressed variants include nt no. 21 C to T, and nt no. 23 G to T. As expected, little variation was observed at the 5′ end of the majority of the most highly detected miRNAs. However, miR-378 (Fig. 4G) and the cardiac muscle-enriched miR-133a are exceptions. As illustrated in Fig. 4C the most frequently recorded miRNA sequence attributed to miR-133a is missing nt no. 1; indeed 53.96±0.46% of all miR-133a reads are missing this nt. This truncation could have a profound effect on target sequence recognition, and therefore the spectrum of target genes regulated. Indeed, DIANA-microT v3.0 (which accepts novel miRNA sequences) [81], [82] predicts 602 target genes for canonical miR-133a and 145 for the 5′ variant (miR-133a(v)), with 90 genes in common. Gelsolin is among the 55 genes predicted to be targeted by miR-133a(v) but not by miR-133a itself. We used the dual-fluorescence miRNA targeting assay [70] to determine the effects of miR-133a and miR-133a(v) on the expression of a reporter gene with a 3′ gelsolin fragment incorporating the more conserved of the two predicted miR-133a(v) target sites. Suppression of the target gene is reported as a decrease in red/green fluorescence intensity ratio in this assay. Fig. 5 shows that miR-133a(v) was more effective at suppressing the mCherry-gelsolin construct than was miR-133a, and was comparable to a siRNA targeted against the mCherry sequence.

Surprisingly, miR-10b exhibited no exact mature sequences (see Table S3B); on closer analysis sequences aligned to miR-10b were found to exhibit shifting in the mature miRNA cleavage position with 12.38±0.77% of the reads including 1 extra nt and 87.39±0.91% 2 extra nts from the pre-miR sequence at the 5′ end (see Fig. S2). This type of 5′ end variation has been attributed to variability in Drosha cleavage position [83]. The 2 extra 5′ nts give the most abundant isomiR the same seed sequence as rno-miR-10a-5p, although the rest of the isomiR sequence corresponds to rno-miR-10b-5p, which has a single internal mismatch compared to rno-miR-10a-5p. Of 148 target sites in 85 genes predicted for rno-miR-10a-5p by DIANA-microT v3.0, 147 are also predicted for the most frequent miR-10b isomiR, reflecting the dominance of the seed region in target recognition. In comparison, only one of the 10 predicted target genes found for canonical rno-miR-10b is shared with the isomiR.

### A Novel Rat miR-1 Analog

**Figure 6:**
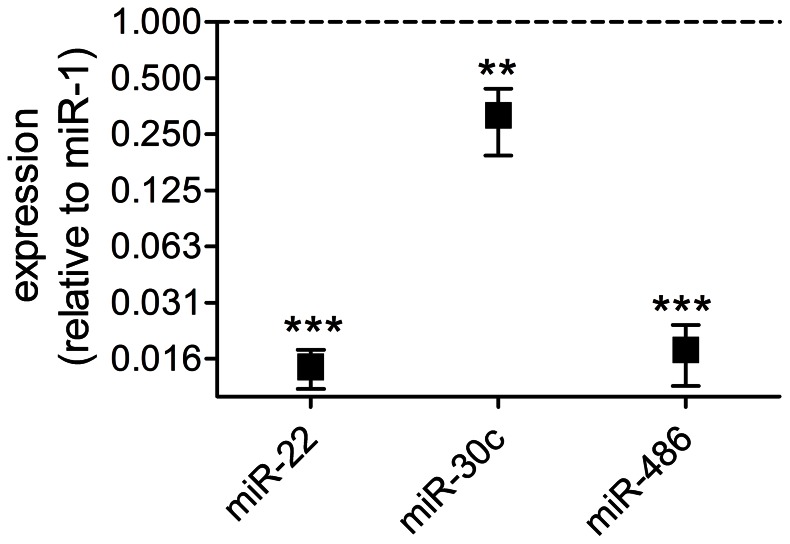
Relative miRNA abundance by TaqMan assay. Expression of miRNAs relative to rno-miR-1 according to 2^−ΔCt^ where ΔCt = Ct – Ct_miR-1_ (mean ± SEM). Ct values were compared by repeated measures ANOVA and Tukey’s multiple comparison test; **p<.01, ***p<.001. Mid-myocardial samples from n = 6 hearts.

It was surprising that miR-1 was only the 28^th^ most abundant miRNA in the sequencing data (Fig. 2) because it is generally considered to be one of the most highly expressed in the heart [84]. It has been demonstrated that observed miRNA abundance can vary significantly between experimental methods [85]. It is possible that preferential cloning of certain sequences or differences in PCR amplification may skew the numbers of reads representing different miRNAs. Indeed, the RNA adapters used can introduce sequencing bias through secondary structure preferences of RNA ligases [86]. The possibility that miR-1 is under-represented in our libraries is supported by the RT-qPCR data shown in Fig. 6 which suggests that, based on relative Ct values, miR-1 is more abundant than miR-22 and miR-486 which are the two most common miRNAs in the deep sequencing data (Fig. 2). Another consideration when comparing sequencing and TaqMan data is the specificity of TaqMan assays for the canonical mature sequence. For example 57% of miR-486 reads in the sample shown in Fig. 4 are 3′ variants that would not be detected by TaqMan because of the 3′ specificity of the looped RT primer used in TaqMan small RNA assays.

**Figure 7:**
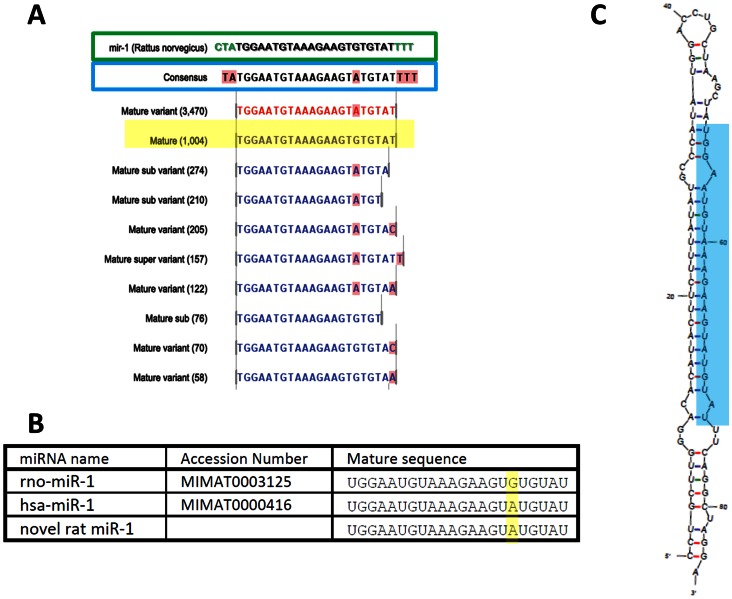
Novel rno-miR-1 sequence as detected by deep sequencing. A: Ten most frequently detected isomiR sequences of miR-1 (rat mature miRNA highlighted in yellow) with expression values in brackets aligned to the published pre-miR sequence (boxed in green; miRBase v18). The consensus sequence (boxed in blue) represents the most prevalent nt aligned at each position (nts highlighted in pink represent variations from the miRBase published mature sequence). B: miR-1 sequences as published in miRBase v18 showing the novel rat miR-1 sequence aligned with the previously reported rat miR-1 and the human sequence (with which it is identical). C: Predicted stem-loop structure (mFold 3.2) of proposed pre-miR deduced from genomic sequence. Mature product highlighted in blue.

Notwithstanding the potentially anomalous measure of abundance, the sequencing data enabled us to observe a variant in the sequence of miR-1; at nt no. 17 an A was frequently present in place of the G in the pre-miR sequence (77.23±1.13% of reads; Fig. 7A). The G to A substitution gives a sequence equivalent to hsa-miR-1 (and mmu-miR-1) which has not been reported before in rat (Fig. 7B). It is unlikely that G could be converted to A by post-transcriptional editing; therefore we investigated the possibility that the rat harbours a second miR-1 gene. Although the most common sequence carrying the A (5′-TGGAATGTAAAGAAGTATGTAT-3′) could not be detected in the latest available build of the *Rattus norvegicu*s genome (Baylor 3.4), the sequence matched two rat whole genome shotgun sequences (gb|AAHX01027125.1|; gb|AABR06028312.1|) which have not been incorporated into this build. Fig. 7C shows the genomic context of this novel miRNA and the predicted stem-loop structure (mFold version 3.2 [87]). This sequence is also present in the rat heart data set of Linsen *et al.* [88]. The existence of two rat miR-1 genes has implications for understanding how this important miRNA is regulated.

### A Novel Rat miRNA

**Figure 8:**
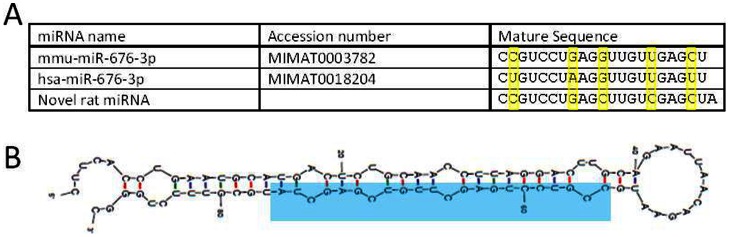
Novel rat miRNA similar to miR-676-3p. A: Alignment of the novel rat miRNA sequence to miR-676-3p sequences as published in miRBase v18. B: Predicted stem-loop structure (mFold 3.2) of proposed pre-miR deduced from genomic sequence. Mature miRNA highlighted in blue.

Analysis of unannotated reads by the miRanalyser utility [89] yielded a putative novel miRNA. This miRNA has a mature sequence similar to mmu-miR-676-3p and hsa-miR-676-3p (Fig. 8A). The genomic context has a predicted stem-loop structure at chrX:88378185-88378273 (Ensembl genome ID ENSRNOG00000040353) (Fig. 8B).

### Cloning and Sequencing of the Rat miR-486 Gene

**Figure 9:**
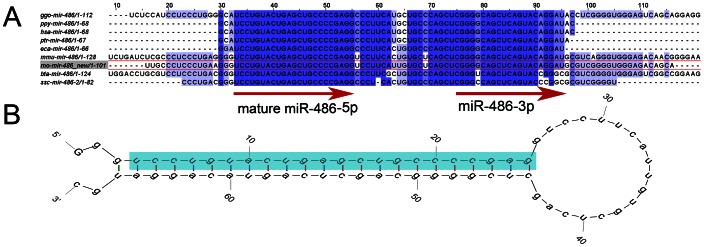
The rat miR-486 gene. A: Alignment of the genomic miR-486 sequence across nine species including rat (highlighted in red). Blue shading indicates percentage sequence identity and the mature and ‘star’ sequences represented by the most common reads are indicated with arrows. B: Predicted stem-loop structure (mFold 3.2) of pre-miR-486 deduced from genomic sequence. Mature miRNA highlighted in blue.

No pre-miR sequence has been published for miR-486 in rat and the mature miRNA sequence is not present in the most recent build of the rat genome (Baylor 3.4). However, alignment of the mouse pre-miR-486 (MI0003493) sequence revealed a potential rat pre-miR-486 sequence on chromosome 16∶73341164 (reverse strand) which is truncated just before the end of the potential miR-486 mature sequence at nt 73341114 by a gap in the genome sequence. A BLAST search revealed two whole genome shotgun sequences spanning this gap, allowing the design of PCR primers that were used to amplify the region from rat genomic DNA. This enabled confirmation of the sequence of the putative miR-486 gene, which contains a stem-loop sequence matching both mature miR-486 reads in the 5p arm and observed ‘star’ sequences from the 3p arm (Fig. 9).

### Transmural Distribution of miRNA Expression

**Figure 10:**
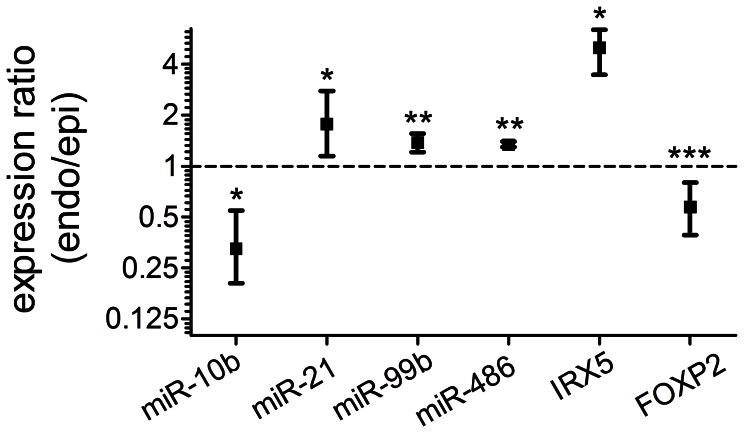
Transmural miRNA expression gradients. Taqman small RNA assays and TaqMan gene expression assays were performed on epicardial and endocardial samples from rat hearts. Endocardial/epicardial expression ratios were determined by analysis of Ct values using REST 2009. The reference genes utilised were miRNAs with stable expression across samples according to BestKeeper: miR-22, miR-30a, miR-30c, miR-30e and miR-100. The reference genes for IRX5 and FOXP2 were GAPDH, HPRT1 and 18s rRNA. Data are mean ± SEM; n = 4 hearts for miRNAs (n = 3 of these for IRX5 and FOXP2). *P<.05; **P<.01; ***P<.001.

Several aspects of cardiomyocyte physiology are known to vary across the left ventricular wall [66], [90]–[111] and the mechanism undoubtedly involves differential gene expression. Transmural expression level is uniform for the vast majority of genes but there are some notable exceptions [112]–[114]. Given the role of miRNAs in regulating gene expression it would be useful to determine the extent of transmural heterogeneity of the expression of miRNAs themselves. In accordance with other gene expression data [112] we found the transmural distribution of miRNA expression to be generally uniform. Nevertheless, differences in relative abundance were observed between endocardium and epicardium for several miRNAs (Tables 1, 2, and 3). Some of the more abundantly detected miRNAs showed some transmural difference that did not reach statistical significance across the three hearts. We selected some of these miRNAs for further investigation by qPCR (TaqMan miRNA assays) in four additional hearts and found statistically significant (p<.05) transmural expression gradients for miR-10b, miR-21, miR-99b and miR-486 (Fig. 10). Transmural expression gradients for IRX5 and FOXP2 were in agreement with a previous report [112]. Deep sequencing shows a transmural gradient (epicardium>endocardium) for miR-10b (Table 2, “grouped on mature”). As discussed above, this group includes no canonical miR-10b, with only 5.6% of reads having the canonical 3′ end (4^th^ and 6^th^ in the list in Fig. S2). This could explain the high Ct values (low abundance) we obtained with the miR-10b TaqMan assay which is designed to be specific for the exact 3′ end of the canonical sequence. Additionally, sequencing analysis identified 8 individual sequences within the miR-10a/b family (unique sequences; Table 3) as having >2 fold detected difference (epicardium>endocardium) within the samples tested.

**Table 1:** Epicardial/endocardial differential expression of exact mature (or mature*) miRNAs (≥ ±1.5 fold difference for miRs of ≥10 raw reads detected in either layer).

Exact mature/*sequences		Epi-cardium		Endo-cardium	
		RPMM		RPMM	
miR name	Fold change	Mean	SEM	Mean	SEM
rno-mir-31	3.00	4.45	2.62	13.36	3.49
rno-mir-326	1.92 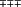	12.52	0.98	24.06	2.03
rno-mir-872	1.80	11.42	2.54	20.61	5.32
rno-mir-1249	1.66	62.40	12.09	103.28	28.92
rno-mir-503*	1.64	5.02	1.45	8.21	0.65
rno-mir-193	1.54	14.14	2.23	21.82	8.28
rno-mir-872*	1.53	51.11	7.20	78.40	14.18
mmu-mir-1983	1.53	8.16	3.02	12.45	2.99
rno-mir-145	1.50	1038.98	188.02	1558.55	346.49
rno-mir-10a	−1.51	1967.41	189.80	1305.65	527.54
rno-mir-9-1//mir-9-3//mir-9-2	−1.52	64.65	13.48	42.62	11.51
rno-mir-208	−1.52	12.33	3.51	8.09	3.41
rno-mir-132	−1.61	18.32	1.35	11.37	2.24
rno-mir-138-2//mir-138-1	−1.66	17.24	2.76	10.36	4.83
rno-mir-210*	−1.69	7.65	1.69	4.53	1.10
rno-mir-100	−1.70	2089.07	827.35	1230.28	597.96
rno-mir-133b	−1.70	15.27	4.06	8.98	2.54
rno-mir-133a*	−1.72	206.39	59.22	120.26	27.43
rno-mir-17-1//mir-17-2	−1.81	10.74	1.68	5.93	0.32
rno-mir-322*	−1.98	20.39	8.69	10.31	5.30
mmu-mir-720	−2.00	23.31	5.27	11.66	1.00
rno-mir-425	−2.25	15.66	3.01	6.96	3.58
hsa-mir-720	−2.30	39.25	8.00	17.09	2.93
rno-mir-374	−2.49	8.51	3.73	3.42	0.66
rno-mir-410	−2.88 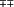	56.31	2.35	19.58	5.31
hsa-mir-4492	−2.92	26.70	10.82	9.14	4.45
rno-mir-200b	−5.88	13.39	10.25	2.28	1.23
rno-mir-547*	−9.73	6.82	3.21	0.70	0.70

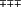
P<0.001,

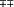
P<0.01, Baggerleys multiple comparison test.

**Table 2:** Epicardial/endocardial differential expression of 'grouped on mature' miRNAs (≥ ±1.5 fold difference for miRs of ≥10 raw reads detected in either layer).

'Grouped on mature' sequences		Epi-cardium		Endo-cardium	
		RPMM		RPMM	
miR name	Fold change	Mean	SEM	Mean	SEM
rno-mir-200b	−4.26	25.92	16.66	6.08	3.31
rno-mir-410	−2.87 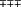	70.10	3.37	24.41	5.32
mmu-mir-690	−2.55	12.73	3.72	5.00	1.39
rno-mir-10b	−2.51 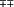	3064.12	431.15	1221.18	441.95
mmu-mir-720	−2.20	27.78	6.87	12.63	1.12
mmu-mir-351	−2.08	11.37	2.93	5.47	0.60
hsa-mir-4485	−2.07	9.81	2.27	4.75	1.81
rno-mir-341	−2.05	7.78	2.10	3.80	2.45
mmu-mir-5115	−1.99	5823.13	1542.50	2930.00	1218.62
rno-mir-384	−1.97	8.73	2.79	4.43	1.46
bta-mir-2478	−1.96	358.54	67.41	182.69	21.31
hsa-mir-720	−1.96	68.26	12.81	34.90	6.30
hsamir-4492	−1.92	43.13	13.29	22.46	9.31
cbr-mir-1	−1.91	10.46	4.84	5.47	1.06
rno-mir-10a	−1.87	30279.72	3036.38	16169.54	6084.64
hsa-mir-1260a	−1.77	278.56	53.99	157.62	42.34
rno-mir-338	−1.77	117.08	1.79	66.29	7.61
mmu-mir-5102	−1.73	11.49	3.65	6.65	1.16
rno-mir-138-2//mir-138-1	−1.71	27.70	5.62	16.18	8.01
rno-mir-100	−1.70	5514.57	2226.60	3245.17	1552.80
rno-mir-132	−1.63	37.90	2.55	23.21	4.82
rno-mir-9-1//mir-9-3//mir-9-2	−1.58	169.45	26.81	107.12	26.08
ssc-mir-186	−1.57	16.66	5.25	10.60	1.13
dre-mir-26b	−1.54	25.55	7.97	16.64	2.29
cte-mir-1	−1.53	6.37	1.86	4.17	1.71
gga-mir-3535	−1.52 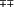	9.46	0.21	6.24	1.83
rno-mir-374	−1.52	64.17	18.59	42.34	7.63
rno-mir-411	−1.50 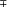	39.14	3.72	26.11	4.25
rno-mir-145	1.50	1537.62	272.14	2300.09	506.26
ola-mir-27d	1.50	6.30	1.14	9.42	3.09
gga-mir-193b	1.51	28.48	5.52	42.86	6.54
mmu-mir-101c	1.56	7.32	2.86	11.39	0.42
rno-mir-326	1.57	37.54	1.72	59.00	5.46
bta-mir-1306	1.63	5.20	1.59	8.45	0.66
rno-mir-877	1.64	8.05	1.21	13.20	1.75
sha-mir-716a	1.75	8.22	0.13	14.37	6.10
rno-mir-674	2.15	5.44	0.81	11.67	1.74
rno-mir-31	2.54	9.37	4.26	23.78	6.64

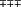
P<0.001.

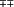
P<0.01,

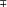
P<0.05 Baggerleys multiple comparison test.

**Table 3:** Epicardial/endocardial differential expression of unique sequences (≥ ±2 fold difference for miRs of ≥100 mean normalised reads detected in either layer). ^
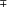
^P<0.05 Baggerleys multiple comparison test.

Unique sequences			Epi-cardium		Endo-cardium	
			RPMM		RPMM	
Sequence	miR name (type)	Fold change	Mean	SEM	Mean	SEM
TACCCTGTAGAACCGAATTTG	rno-mir-10b(P)	−2.99	140.45	22.37	46.95	18.79
CTGGACGCGAGCCGGGCCCTT	mmu-mir-5115(MSupV)	−2.82	753.60	405.10	267.59	109.66
TACCCTGTAGAACCGAATTTGT	rno-mir-10b(MSub/Sup)	−2.54	2108.85	325.91	829.98	294.94
ATCCCACTTCTGACACCA	bta-mir-2478(MSub)	−2.45^ 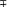 ^	196.81	33.86	80.26	6.23
CCGCGTCGGCGGTTCCC	nlo-mir-125(PV)	−2.43	138.79	42.98	57.12	25.49
ACCCTGTAGAACCGAATTTGT	rno-mir-10b(MSub/Sup)	−2.42	119.77	21.71	49.43	15.33
TACCCTGTAGATCCGAATTTGA	rno-mir-10a(MSubV)	−2.22	325.04	20.21	146.52	55.43
CTGGACGCGAGCCGGGCCCTTCCC	mmu-mir-5115(PV)	−2.21	147.76	43.14	67.01	26.00
TCGTACCGTGAGTAATAATGC	rno-mir-126(MSub)	−2.17	821.18	347.35	378.83	99.04
TACCACAGGGTAGAACCACGGAA	rno-mir-140(M*SupV)	−2.16	348.57	66.62	161.25	22.11
ACCCTGTAGATCCGAATTTG	rno-mir-10a(MSub)	−2.14	272.77	37.62	127.17	50.55
ACCCTGTAGATCCGAATTTGT	rno-mir-10a(MSub)	−2.11	1779.84	246.64	842.11	327.87
CTGGACGCGAGCCGGGC	mmu-mir-5115(MSubV)	−2.10	508.53	119.83	241.60	100.94
TACCCTGTAGAACCGAATTTGTG	rno-mir-10b(MSub/Sup)	−2.09	192.96	12.82	92.16	32.52
TACCCTGTAGATCCGAATTT	rno-mir-10a(P)	−2.08	349.57	32.37	168.25	69.42
CTGGACGCGAGCCGGGCCCTTCC	mmu-mir-5115(PV)	−2.05	188.96	62.04	92.07	34.57
CTGGACGCGAGCCGGGCCCTTC	mmu-mir-5115(MSupV)	−2.04	775.70	233.89	380.53	157.79
AACCCGTAGATCCGAACTTGTGA	rno-mir-100(MSupV)	−2.01	768.45	357.07	382.85	189.43
TGTAAACATCCTCGACTGGAAGCG	rno-mir-30a(MSupV)	2.06	118.32	23.96	243.52	139.23
TCCTGTACTGAGCTGCCCCGG	bta-mir-486(MSubV)	2.39	133.14	28.86	318.16	181.42
GGGGGGCCCAAGTCCTTCTGATCGAGGCCC	mmu-mir-5105(P)	2.93	73.61	16.57	215.65	93.91
CATTCAACGCTGTCGGTGAGT	rno-mir-181a-2//mir-181a-1(MSub)	40.70	6.08	0.93	247.35	243.97
CATTCAACGCTGTCGGTGAG	rno-mir-181a-2//mir-181a-1(MSub)	63.29	2.65	0.93	167.78	165.76

### Prediction of Signalling Pathway Targets

**Figure 11:**
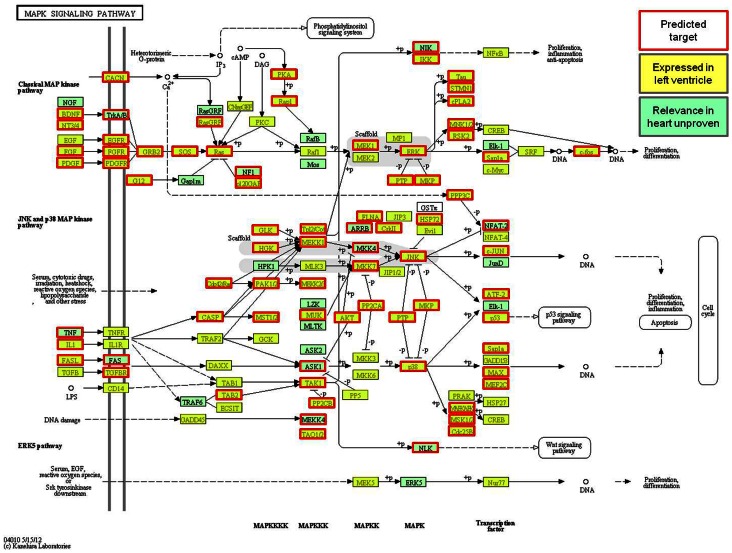
Pathway analysis of MAP Kinase signaling (Kegg pathway). Highlighted genes are targets predicted by 2 or more algorithms of the top 16 highest detected miRNAs (boxed in red; P = 1.86×10^−6^; 75 targets) showing overlap with genes expressed in the heart (highlighted; expression data 9 SD rat left ventricular myocardium samples from 2 microarray experiments, (GSE6943; GSM160095–100 & GSE6880; GSM158589–91; http://www.ncbi.nlm.nih.gov/gds).

To reveal the pathways most likely to be significantly regulated by miRNAs, pathway analysis was performed based upon the top 16 most highly detected miRNA sequences (>10,000 RPMM) utilizing DIANA LAB (loose, strict and Beta algorithms as well as TargetScan5 and Pictar [115] combined with TargetScan 6.0 (including poorly conserved sites – Total context score ≤-0.1) and Micro-org individual predictions (mirSVR score ≤ -0.3). Excluding targets which only appeared on one of the 7 prediction lists, the resultant 2808 genes were compared with a list of genes expressed in heart (as identified from 9 Sprague-Dawley rat left ventricular myocardium samples from 2 microarray experiments published on GEO (GSE6943; GSM160095-100 & GSE6880; GSM158589-91; http://www.ncbi.nlm.nih.gov/gds)) resulting in 1624 potential targets expressed in and therefore most relevant to heart. Of the significantly targeted pathways the most relevant to cardiac physiology and pathophysiology were the Ubiquitin-mediated Proteolysis pathway (Benjamini corrected P = 7.12×10^−7^; 46 targets), Mitogen-Activated Protein Kinase pathway (MAPKinase; P = 1.86×10^−6^; 75 targets; Fig. 11), Regulation of Actin Cytoskeleton (P = 2.29×10^−4^; 56 targets), Wnt pathways (P = 3.45×10^−4^; 42 targets), Calcium Signaling (P = 5.01×10^−4^; 49 targets), Gap junctions (P = 6.43×10^−4^; 27 targets) and Arrhythmogenic Right Ventricular Cardiomyopathy pathway (ARVC; P = 0.047; 20 targets).

Further analysis of a shortlist of genes important in contraction, electrical activity, Ca^2+^ signalling, cell communication, ion homeostasis and gene expression showed potential targeting of components contributing to the generation of I_Na_, I_Ca,L_, I_Kur_, I_to_, I_KATP_, I_NaCa_, I_f_, I_Kp_ as well as SR Ca^2+^ re-uptake and contraction (see Table S6).

## Discussion

### Value of Deep Sequencing Data

The rat is widely used as a model of heart disease and the importance of miRNAs in heart disease is by now well recognised. Therefore it is important to know as much as possible about miRNA expression in the rat heart. Deep sequencing yields information over and above that which can be obtained from microarrays or qPCR, in that the miRNAs that can be detected are not pre-designed into the method. Thus it provides the ability to detect novel sequences, including novel miRNAs and novel isomiRs of known miRNAs. This advantage is well illustrated in the present study.

Our data provide some interesting comparisons with the few previous next-generation sequencing studies in the heart. Linsen et al. [88] analysed rat heart using the SOLiD™ platform (Life Technologies) and detected 180 miRNAs in common with our study. There were 206 miRNAs detected in the present study that were not found in rat heart by Linsen et al. and 357 miRNAs reported in heart by Linsen et al. did not appear in our data. However of the top 20 miRs detected by Linsen et al., 10 were also highly detected in the present study. More recently Vacchi-Suzzi et al. [116] also investigated the expression of miRNA in rat left ventricle by next generation sequencing using HiSeq in conjunction with TruSeq Small RNA Sample Preparation Kits (Illumina). Vacchi-Suzzi et al. identified 636 rno- and/or mmu-miRs including expression of mmu-miR-486 as well as co-incidence of 13 of their top 20 highest detected miRNAs with the present study. In the 3 rat studies 5 common miRNAs were highly detected; rno-miR-26a, 30a, 30c, 30d & 30e.

In mouse heart 3 groups have investigated the miRNA expression profile ([61], [117], [118] using next generation technologies (Illumina, HiSeq 2000 and SOLiD™ platforms respectively). Of the Top 20 miRNAs detected in each mouse study 6 were detected in all 3 (mmu-miR-1, 126, 378, 26a, 125b & 133a). Thirteen of the top 20 most frequently detected miRNAs in our data were also in the top 20 of Rao et al.’s data [117], while 7 were also highly detected in the Humphreys et al. study [61] and 12 were also highly detected by Yang et al. [118]. The differences between studies are likely attributable to the combined effects of different species, tissue types (left ventricular wall *vs* whole heart), sequencing technologies and data analysis parameters. Additionally, given that the myocardium is not composed solely of cardiomyocytes it is unlikely that these profiles are the product of only this cell type; indeed Humphreys et al. identified 143 miRNAs which were expressed in either mouse left ventricular or HL-1 cell samples but not in both [61]. As the mid-myocardium (as used in the present study) also contains fibroblasts and vascular endothelial cells, the contribution of these cell types to the expression of miRNA needs to be considered; miR-21 (which we detected as 30th highest) has been shown to be expressed up to 4-fold higher in cardiac fibroblasts than myocytes [119] as well as being highly detected in various vascular beds [54], [55], [120]. However as some miRNAs are known to be highly exported [121], [122], especially in disease states [5], the cellular origin of the sequence may be of less importance than where they are functionally active.

### IsomiR Expression

Variability at the 5′ end is likely to have a significant effect on targeting because nts 2–8 form the seed region of the miRNA, which has a considerable contribution to target determination [1]. Therefore the ability to detect isomiRs and distinguish them from canonical miRNAs will enhance our understanding of the role of miRNAs in health and disease. A good case in point is miR-133a, of which more than half of the reads in our dataset were of a 5′ variant isomiR (miR-133a(v)). Furthermore there is evidence from miRBase for a similar proportion of miR-133a(v) *vs* miR-133a expression in human. Since miR-133a is enriched in the heart and dysregulated in heart disease [123]–[128], some changes in gene expression in heart disease may be attributable to changes in miR-133a(v). Genes predicted by DIANA-microT v3.0 to be targets of miR-133a(v) but not canonical miR-133a include gelsolin and KCNIP2 (KChIP2). Gelsolin is upregulated and promotes apoptosis in cardiomyopathy [129]. If a substantial proportion of all miR-133a isomiRs expressed in normal myocardium is miR-133a(v) (as suggested by our data) then the downregulation of this variant could contribute to the upregulation of gelsolin in heart disease. A precedent for the possibility of miR-133a isomiR-specific targeting has already been established [61]. Additionally, KCNIP2 (an accessory protein that modulates several cardiac ion channels and is involved in cardiac arrhythmia and heart failure [130]) was downregulated by overexpression of a miR-133a genomic precursor in mouse heart [131]. This was a surprising finding because KCNIP2 is not a predicted target of miR-133a. It is plausible that KCNIP2 was downregulated by miR-133a(v) if a substantial proportion of the genomic precursor were processed to form this isomiR.

IsomiRs with variations at the 3′ end are less likely to differ in terms of genes targeted because they do not alter the seed region. However, 3′ variation may affect certain target sites which rely on complementarity at the 3′ end, with potentially important physiological consequences.

### Novel Rat miRNAs and miRNA Genes

The importance of miR-1 in the heart is by now well recognised [132]. Manipulation of miR-1 expression in the rat heart may be a useful strategy for further investigating the physiological role of miR-1. However, such studies could have been confounded by the unknown existence of a second miR-1 gene. Our discovery of the second gene is therefore critical for miR-1 expression studies in the rat.

The putative novel miRNA reported in Fig. 8 has potential implications for the expression of its target genes. DIANA-microT v3.0 predicts 25 target sites in 7 genes for this miRNA sequence. The putative target genes are ADAMTS13 (von Willebrand factor-cleaving protease), SLC6A3 (Sodium-dependent dopamine transporter), PCNT (Pericentrin), MBD3 (Methyl-CpG-binding domain protein 3), ICOSLG (ICOS ligand precursor (B7 homolog 2)), FOXK2 (Forkhead box protein K2), and FOXK1 (Forkhead box protein K1).

Small et al. [72] reported enrichment of miR-486 in cardiac and skeletal muscle, and presented evidence for miR-486 as a downstream mediator of the actions of serum response factor, myocardin-related transcription factor-A and MyoD in muscle cells and a potential modulator of PI3K/Akt signalling in rat neonatal cardiomyocytes. Despite the likely importance of this miRNA in rat muscle, rat miR-486 does not appear in the current version of miRBase. This is presumably because the complete miR-486 gene is not in the rat genome database. We used PCR to read into this gap and determined the sequence of the pre-miR (Fig. 9). This information should be useful for future studies of miR-486 in rat models.

### Transmural Expression Gradients

TargetScan 6.2 predicts 307 conserved targets for hsa-miR-21, 56 for hsa-miR-99b and 154 for hsa-miR-486-5p. FOXP2 is a predicted target of miR-21; its expression is higher in epicardium than endocardium [112] (Fig. 10), which is the opposite to the gradient for miR-21 expression. This is consistent with miR-21 influencing transmural FOXP2 expression.

Calcineurin Aα (PPP3CA) is a predicted target of miR-21 and miR-99b (TargetScan 6.2) and is expressed more in epicardium than endocardium [113]. Calcineurin is a calmodulin-activated serine/threonine protein phosphatase that mediates cardiac hypertrophy progressing to heart failure [133] and electrical remodelling [134]. In relation to left ventricular transmural physiology, calcineurin downregulates expression of the Na^+^ channel Na_v_1.5 (SCN5A) and the gap junction proteins Cx40 (GJA5) and Cx43 (GJA1) [135], all of which are expressed in a transmural gradient opposite to that of calcineurin Aα [100], [112]–[114]. Expression of the K^+^ channel K_v_4.2 (KCND2) [136], the sarcoplasmic reticulum Ca^2+^ pump SERCA2 (ATP2A2) [137] and the Na^+^-Ca^2+^ exchanger NCX1 (SLC8A1) [137] is upregulated by calcineurin, and the transmural expression pattern of these genes parallels that of calcineurin [112], [113]. Thus, at the level of gene expression, there are consistencies that point toward a role of calcineurin Aα in determining transmural gradients in cardiac physiology. Nevertheless there may be non-transcriptional effects that make the picture more complicated. Calcineurin phosphatase activity is actually higher in endocardium than epicardium, secondary to higher intracellular [Ca^2+^] [138]. Calcineurin may have non-transcriptional inhibitory effects on SERCA2 [139] and NCX1 [140], [141] activity. The fast transient outward potassium current (I_to,f_) was increased by overexpression of constitutively active calcineurin in cultured neonatal rat ventricular myocytes [136] but reduced in calcineurin-overexpressing transgenic (TG) mice [142]. The β isoform of calcineurin A is also expressed in heart, uniformly across the left ventricular wall [138]. The relative importance of the two isoforms is not clear. Inactivation of the calcineurin Aβ gene in mice caused an 80% decrease in calcineurin enzymatic activity in the heart [143], suggesting that Aβ is the dominant calcineurin A isoform. On the other hand, silencing of calcineurin Aα was more effective at reducing SERCA2 expression than calcineurin Aβ silencing in neonatal rat cardiomyocytes [137]. Thus a role for miR-21 and/or miR-99b in influencing transmural physiology is an intriguing possibility but requires further study. It should also be mentioned that the determination of transmural calcineurin Aα expression was made in myocardial samples containing all cell types [113]. The above discussion pertains to cardiomyocytes but a transmural expression gradient in other cell types cannot be excluded. miR-21 is abundant in cardiac fibroblasts [119], in which calcineurin may be involved in regulating cell proliferation [144], [145]. miR-21 is enriched in endothelial cells [54], [55], [120], in which it mediates the endothelial-to-mesenchymal transition [146] and angiogenesis [147], [148]. Enrichment of miR-99b was found in cardiac valves [116], although there is no available evidence for a role of miR-99b in valve disease.

NCX1 (SLC8A1) is important for cardiomyocyte Ca^2+^ extrusion. Its higher expression in epicardium *vs* endocardium [113] is consistent with high endocardial intracellular [Ca^2+^] [138]. NCX1 is a predicted target of miR-486 (TargetScan 6.2), which we found to be higher in endocardium than epicardium (Fig. 10). Therefore miR-486 could conceivably influence transmural differences in intracellular Ca^2+^ handling.

IRX5 is a predicted target of miR-486, as discussed above. IRX5 negatively regulates K_v_4.2 K^+^ channel expression, and the IRX5 transmural expression gradient (higher in endocardium) [112] (Fig. 10) is responsible for the expression gradient (higher in epicardium) of Kv4.2 in the heart [76], [77]. miR-486 may be able to influence IRX5 expression but its own expression gradient tends in the opposite direction for it to be responsible for the transmural gradient of IRX5.

DIANA-microT v3.0 predicts 147 targets in 85 genes for the most abundant rno-miR-10b isomiR (Fig. S2). Among these, the transcription factor TBX5 is expressed in a transmural gradient that changes with heart development [149].

### Signalling Pathways

Within the Ubiquitin-mediated Proteolysis pathway the E1 enzyme UBLE1A and 14 of the 22 heart-expressed E2 enzymes are predicted to be targeted by the 16 most highly expressed miRNAs. Six of the 11 heart-expressed HECT type E3 enzymes, 3 out of 5 heart expressed U-box type E3 enzymes, 6 of 13 heart expressed single RING-finger type E3 enzymes and 10 of 36 heart expressed proteins within the multi subunit RING-finger type E3 complex are predicted targets. Recent studies have described how ubiquitination in the heart is upregulated in common cardiac diseases, including cardiac hypertrophy, heart failure, ischemia-reperfusion injury, and diabetes (for review see [150]). The overwhelming nature of the predicted targeting of the Ubiquitin-mediated Proteolysis Pathway suggested by the expression profile of our samples may reflect the importance of keeping this mechanism under tight control in normal tissue.

Within the MAP Kinase pathway 55 of the 98 heart expressed gene families (representing a total of 75 targets in 151 heart genes) were predicted as potential targets (Fig. 11). Interestingly the Ras gene family within this pathway was predicted to be targeted by 14 of the top 16 highest detected miRNAs; this family of genes as a part of the Ras/Raf-1/MEK/ERK cascade is generally regarded as playing a highly significant role in cardiac hypertrophy, myocardial cell death and myocardial remodelling [151].

Another highly targeted pathway, Wnt signalling, which is required for basic developmental processes such as control of asymmetrical cell division, remodelling of the cytoskeleton and cell adhesion [152], [153] is likely to be of importance in the heart especially in relation to the Wnt5 receptor, as Frizzled, and downstream proteins PLC, CAMKII and Calcineurin are all implicated in NFAT transcriptional changes associated with hypertrophy [154], [155]. All of our top 16 highest detected miRNAs are predicted to target at least one of the genes encoding proteins in the Wnt5 pathway.

### Conclusion

We have used deep sequencing to measure miRNA expression across the left ventricular wall of the adult rat heart. This has highlighted the complexity of the *in vivo* miRNA profile, as suggested by previous studies of cardiomyocyte cells [61]. Many miRNAs not previously reported in rat were detected and for each miRNA a range of isomiRs was present. Thorough characterisation of the normal pattern of miRNA expression is an important basis from which to study the role of miRNAs in regulating gene expression in the heart. Whilst transmural expression was uniform for most miRNAs, the gradients found for several miRNAs are intriguing and suggest potentially important roles in heart physiology.

## Supporting Information

Figure S1 **miRNA library construction from three rat heart samples assessed using the Bioanalyser 2100.**
(PDF)

Figure S2 **Expression profile of rno-miR-10b isomiRs in mid-myocardium.**
(PDF)

Table S1 **Sequences of PCR primers.**
(PDF)

Table S2 **Oligonucleotide sequences of pSM30 inserts utilized to express artificial pre-miRs.**
(PDF)

Table S3 **MiRNA reads annotated to previously characterised rat sequences.**
(PDF)

Table S4 **MiRNA annotated to sequences not previously characterised in rat but identified in other species.**
(PDF)

Table S5 *strand miRNA annotated to previously characterised sequences.
(PDF)

Table S6 **Shortlist of genes relevant to cardiac contractile function predicted to be targeted by the top 16 highest detected miRNAs.**
(PDF)
