# Supplementary material for: Distinctive Profile of IsomiR Expression and Novel MicroRNAs in Rat Heart Left Ventricle
Source: PLoS One. 2013 Jun 14;8(6):e65809. doi: 10.1371/journal.pone.0065809 (PMC3683050; doi:10.1371/journal.pone.0065809)
Supplement: Figure S1 — miRNA library construction from three rat heart samples assessed using the Bioanalyser 2100. (PDF) [file pone.0065809.s001.pdf]

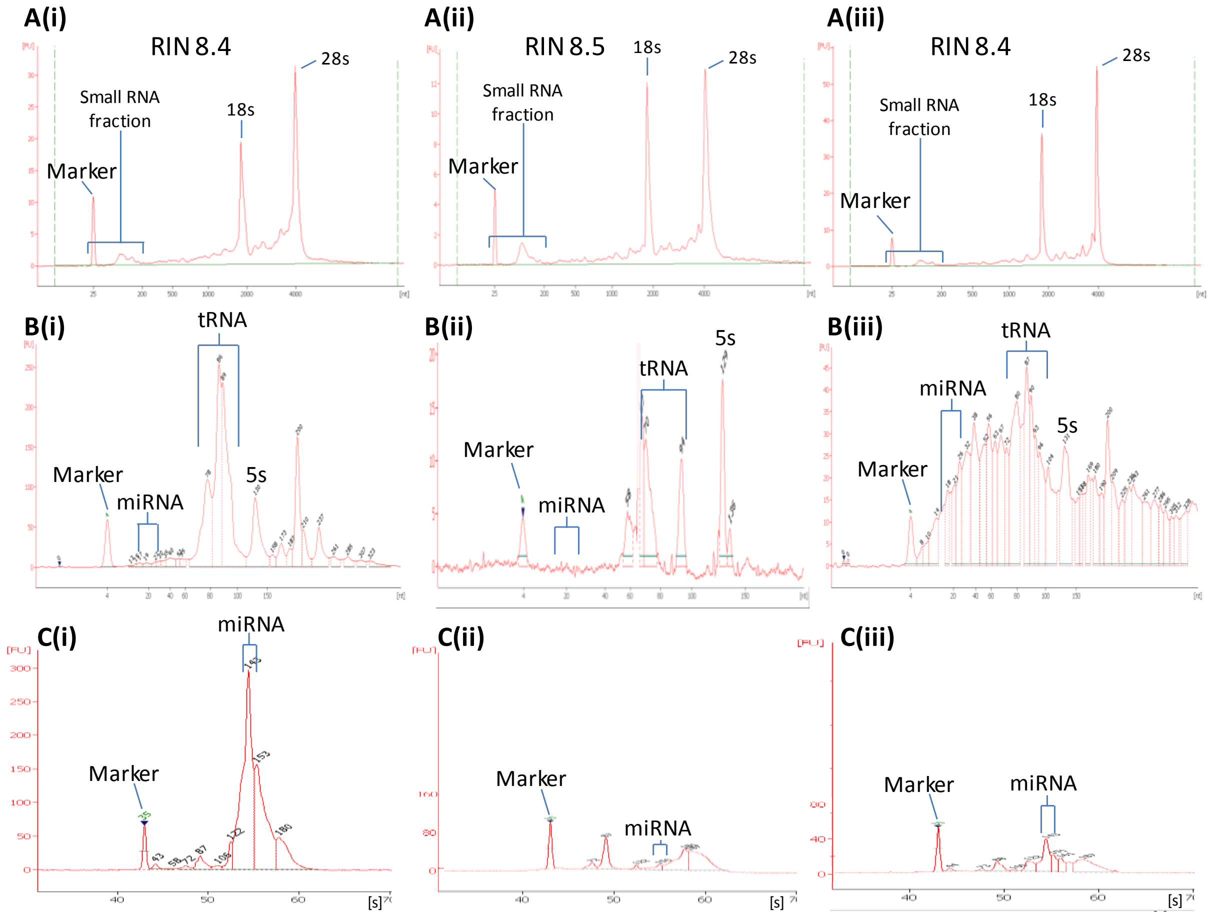

Fig S1. miRNA library construction from three rat heart samples **(i-iii)** assessed using the Bioanalyser 2100.

A) Typical RNA nano chip electropherograms of RNA samples with high quality as established by RIN (>8.0).

B) Small RNA chip electropherograms of the samples in **A** illustrating in: **(i)** the presence of miRNA (and other small RNA species; note the dominance of the triple peaked tRNA complex); **(ii)** the relative absence of small RNA species and complete absence of miRNA (the masked peak at 68nt is a movement artefact); **(iii)** the over abundance of small RNA species which almost entirely mask the tRNA and 5S peaks. The electropherograms are normalised to the marker amplitude.

C) High sensitivity DNA chip electropherograms of the libraries prepared from the samples in **B** illustrating in: **(i)** the presence a dominant peak close to the predicted length of 147nt for the product amplified from miRNA cDNA (143nt peak concentration 1,123.78 pg/µl; discrepancy of measured peak due to sizing inaccuracies of the chip; ref the manual for % inaccuracies); **(ii)** 147nt peak is absent in this sample; **(iii)** small peak close to 147nt (143nt peak concentration 116.88 pg/µl). The electropherograms are normalised to the marker amplitude.
