# Supplementary material for: Distinctive Profile of IsomiR Expression and Novel MicroRNAs in Rat Heart Left Ventricle
Source: PLoS One. 2013 Jun 14;8(6):e65809. doi: 10.1371/journal.pone.0065809 (PMC3683050; doi:10.1371/journal.pone.0065809)
Supplement: Figure S2 — Expression profile of rno-miR-10b isomiRs in mid-myocardium. (PDF) [file pone.0065809.s002.pdf]

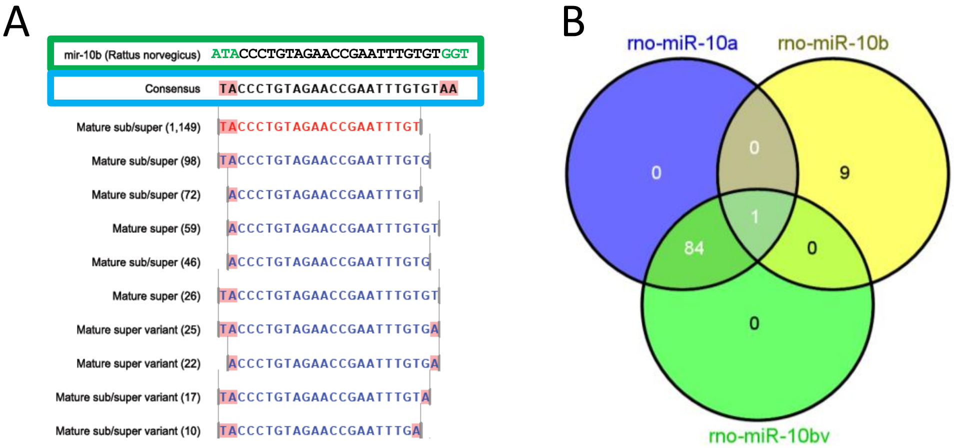

C

miRNA name	Accession Number	Sequence
rno-miR-10b	MIMAT0000783	CCCUGUAGAACCGAAUUUGUGU
rno-miR-10bv		UACCCUGUAGAACCGAAUUUGU
rno-miR-10a	MIMAT0000782	UACCCUGUAGAUCCGAAUUUGUG
mmu-miR-10b	MIMAT0000208	UACCCUGUAGAACCGAAUUUGUG

Fig S2 Expression profile of rno-miR-10b isomiRs in mid-myocardium.

A) Ten most highly detected miR-10b isomiR sequences with expression values in brackets aligned to the published pre-miR sequence (boxed in green; miRBase v18). The consensus sequence (boxed in blue) represents the most prevalent nucleotide aligned at each position (nucleotides highlighted in pink represent variations from the miRBase published mature sequence). **B)** Target genes for mature (rno-miR-10b), most prevalent isomiR (rno-mir-10bv) and rno-miR-10a predicted by DIANA-microT v3.0. **C)** Sequence alignments for miR-10a&b and most prevalent variant.
