# Supplementary material for: Distinctive Profile of IsomiR Expression and Novel MicroRNAs in Rat Heart Left Ventricle
Source: PLoS One. 2013 Jun 14;8(6):e65809. doi: 10.1371/journal.pone.0065809 (PMC3683050; doi:10.1371/journal.pone.0065809)
Supplement: Table S1 — Sequences of PCR primers. (PDF) [file pone.0065809.s003.pdf]

Primer annotation	Primer direction	Primer sequence
pre-miR-486F	Forward	TAGTGCTGTGCATGGGGCA
pre-miR-486R	Reverse	AGGTGTGCATCAGGGCAGA
hGSNFg2	Forward (outer)	TCCCCTCGGGAGGCGAGTTC
hGSNRg2	Reverse (outer)	ACACCTGGCACTGCCCCCTT
hGSN3FXhoI	Forward (inner)	CAAGGACTCGAGATCGAAGAGGTTCCTGGTG
hGSN3RBamHI	Reverse (inner)	AGGAAGGGATCCCTGGTCCCAGGTGTCC

Table S1. Sequences of PCR primers. The top two were used for amplifying the miR-486 stem-loop sequence from rat genomic DNA. The other four were used for nested amplification of part of the gelsolin gene from human genomic DNA. The inner primers incorporate restriction sites for subcloning.
