# Supplementary material for: Distinctive Profile of IsomiR Expression and Novel MicroRNAs in Rat Heart Left Ventricle
Source: PLoS One. 2013 Jun 14;8(6):e65809. doi: 10.1371/journal.pone.0065809 (PMC3683050; doi:10.1371/journal.pone.0065809)
Supplement: Table S2 — Oligonucleotide sequences of pSM30 inserts utilized to express artificial pre-miRs. (PDF) [file pone.0065809.s004.pdf]

miR-133a

AGCGAAGCTGGTTGAAGGGGACCAAATAGTGAAGCCACAGATGTA<u>TTTGGTCCCCTTCAACCAGCTG</u>

|||||||||||||||||||||||||||||||||||||||||||||||||||||||||||||||

TTCGACCAACTTCCCCTGGTTTATCACTTCGGTGTCTACATAAACCAGGGGAAGTTGGTCGACACGG

miR-133a(v)

5′ AGCGCCAGCTGGTTGAAGGGGACCAATAGTGAAGCCACAGATGTA<u>TTGGTCCCCTTCAACCAGCTGT</u> 3′

|||||||||||||||||||||||||||||||||||||||||||||||||||||||||||||||

3′ GGTCGACCAACTTCCCCTGGTTATCACTTCGGTGTCTACATAACCAGGGGAAGTTGGTCGACAACGG 5′

siR-mCh

5′ AGCGTCCTACAACGTCAACATCAAGTTAGTGAAGCCACAGATGTA<u>ACTTGATGTTGACGTTGTAGGC</u> 3′

|||||||||||||||||||||||||||||||||||||||||||||||||||||||||||||||

3′ AGGATGTTGCAGTTGTAGTTCAATCACTTCGGTGTCTACATTGAACTACAACTGCAACATCCGACGG 5′

NTC

5′ AGCGCGACGTTCGAACTTACATAACTTAGTGAAGCCACAGATGTA<u>AGTTATGTAAGTTCGAACGTCA</u> 3′

|||||||||||||||||||||||||||||||||||||||||||||||||||||||||||||||

3′ GCTGCAAGCTTGAATGTATTGAATCACTTCGGTGTCTACATTCAATACATTCAAGCTTGCAGTACGG 5′

Table S2. Oligonucleotide sequences of pSM30 inserts utilized to express artificial pre-miRs for canonical miR-133a, variant (miR-133a(v)), siRNA against mCherry (siR-mCh) and a random non-targeting negative control sequence (NTC). 'Mature' sequence underlined.
