# Supplementary material for: Distinctive Profile of IsomiR Expression and Novel MicroRNAs in Rat Heart Left Ventricle
Source: PLoS One. 2013 Jun 14;8(6):e65809. doi: 10.1371/journal.pone.0065809 (PMC3683050; doi:10.1371/journal.pone.0065809)
Supplement: Table S3 — MiRNA reads annotated to previously characterised rat sequences. (PDF) [file pone.0065809.s005.pdf]

Table S3A

Mature Sequence	miRNA name	Exact Mature Normalised (RPMM) Mean	SEM	Grouped on Mature Normalised (RPMM) Mean	SEM	Exact/grouped on Mature % Mean	SEM
AAGCTGCCAGTTGAAGAACTGT	mir-22	148238.18	13770.35	159311.54	14796.14	93.04	0.35
TTTGGTCCCCTTCAACCAGCTG	mir-133a	5269.27	206.87	107335.09	11328.46	5.05	0.66
TGAGATGAAGCACTGTAGCTCA	mir-143	8120.06	1110.82	98836.06	10986.97	8.19	0.53
TTCAAGTAATCCAGGATAGGCT	mir-26a	38359.97	2731.24	54641.40	3784.37	70.18	0.51
TGTAAACATCCTCGACTGGAAG	mir-30a	1489.73	106.30	44019.05	5132.14	3.43	0.21
TTCACAGTGGCTAAGTTCTGC	mir-27b	6941.90	353.27	25192.92	2064.12	27.75	1.46
TGTAAACATCCCCGACTGGAAG	mir-30d	820.48	53.46	24748.18	1439.78	3.31	0.08
AACATTCAACGCTGTCGGTGAGT	mir-181a-2//mir-181a-1	12580.31	2099.34	24724.26	4438.24	51.09	0.59
ACTGGACTTGGAGTCAGAAGG	mir-378	5202.99	311.62	24172.32	931.20	21.53	1.07
TGAGGTAGTAGATTGTATAGTT	let-7f-1//let-7f-2	11292.76	1192.64	19963.82	1967.89	56.49	0.61
TACCCTGTAGATCCGAATTTGTG	mir-10a	1522.37	617.01	18976.58	7830.50	8.18	0.41
TGTAAACATCCTACACTCTCAGC	mir-30c-1//mir-30c-2	2024.09	185.41	18179.44	1193.34	11.10	0.40
CAACGGAATCCCAAAAGCAGCTG	mir-191	12864.94	2765.66	16154.44	2717.93	78.51	3.35
TGTAAACATCCTTGACTGGAAG	mir-30e	207.02	56.50	15686.25	4231.95	1.32	0.03
TGAGGTAGTAGGTTGTATAGTT	let-7a-1//let-7a-2	4522.74	401.85	10429.11	704.24	43.26	1.12
TCCCTGAGACCCTTTAACCTGTGA	mir-125a	1409.33	145.27	9204.24	2439.45	18.27	5.58
TATTGCACTTGTCCCGGCCTG	mir-92a-1//mir-92a-2	77.36	11.13	9118.65	872.27	0.84	0.05
TCTCCCAACCCTTGTACCAGTG	mir-150	3623.46	318.22	8732.43	857.34	41.59	1.01
TAGCACCATCTGAAATCGGTTA	mir-29a	2097.10	175.73	8057.77	694.94	26.04	0.36
TGAGGTAGTAGGTTGTATGGTT	let-7c-1//let-7c-2	3902.94	252.83	6720.77	484.81	58.13	0.40
TCGTACCGTGAGTAATAATGCG	mir-126	2066.73	215.82	6048.59	71.11	34.10	3.17
TGAGGTAGTAGTTTGTGCTGTT	let-7i	2244.67	184.37	5603.70	521.41	40.13	0.45
TTCAAGTAATTCAGGATAGGT	mir-26b	644.43	58.36	5267.87	724.73	12.38	0.55
TACAGTACTGTGATAACTGAA	mir-101a	268.49	35.85	4955.80	688.92	5.43	0.14
TGGAATGTAAAGAAGTGTGTAT	mir-1	734.44	110.22	4696.77	519.27	15.53	0.75
TCCCTGAGACCCTAACTTGTGA	mir-125b-1//mir-125b-2	1883.49	145.60	4422.85	512.99	44.09	6.96
CACCCGTAGAACCGACCTTGCG	mir-99b	1374.34	533.26	4399.66	1692.03	31.16	0.11
TAGCAGCACGTAAATATTGGCG	mir-16	3120.54	822.49	4080.76	964.10	75.59	1.86
CAAAGAATTCTCCTTTTGGGCT	mir-186	1216.10	43.68	2894.01	43.85	42.01	1.25
TAGCTTATCAGACTGATGTTGA	mir-21	1118.75	149.33	2780.89	278.42	39.96	1.44
ATCACATTGCCAGGGATTACC	mir-23b	348.58	41.36	2759.72	188.35	12.58	0.88
TGAGGTAGTAGGTTGTGTGGTT	let-7b	923.27	81.38	2425.99	206.95	38.04	0.19
TGTAAACATCCTACACTCAGCT	mir-30b	1970.32	114.45	2169.56	132.14	90.85	0.62
ATCACATTGCCAGGGATTTCC	mir-23a	773.87	83.51	2078.17	234.88	37.49	2.80
TTCACAGTGGCTAAGTTCCGC	mir-27a	228.48	38.96	2027.89	93.73	11.15	1.38
AGAGGTAGTAGGTTGCATAGTT	let-7d	971.71	83.25	1960.88	129.34	49.44	1.02
AGCAGCATTGTACAGGGCTATGA	mir-103-2//mir-103-1	635.49	85.28	1909.10	153.32	33.04	1.91
TCGAGGAGCTCACAGTCTAGT	mir-151	952.03	64.38	1679.01	136.47	56.83	0.95
AACCCGTAGATCCGAACTTGTG	mir-100	794.00	293.99	1678.78	580.35	46.14	1.90
AACATTCAACCTGTCGGTGAGT	mir-181c	933.22	161.90	1665.99	276.02	55.92	0.81
GTCCAGTTTTCCCAGGAATCCCT	mir-145	1038.71	127.47	1457.51	186.21	71.37	0.87
CAGTGCAATGTTAAAAGGGCAT	mir-130a	428.33	117.42	1318.86	212.95	31.41	3.34
CATTGCACTTGTCTCGGTCTGA	mir-25	680.14	95.13	1317.04	151.27	51.42	1.97
TTAAGACTTGCAGTGATGTTT	mir-499	772.71	91.17	1238.05	163.73	62.68	1.06
AACATTCATTGCTGTCGGTGGGT	mir-181b-1//mir-181b-2	265.63	8.35	1067.70	59.36	24.95	0.66
TTTGGTCCCCTTCAACCAGCTA	mir-133b	10.96	4.09	1029.13	110.52	1.05	0.41
TGAGAACTGAATTCCATGGGTT	mir-146a	298.40	13.47	904.20	53.36	33.07	0.88
CAGCAGCAATTCATGTTTTGGA	mir-322	493.59	18.29	878.40	29.99	56.19	0.50
TAGCACCATTTGAAATCGGTTA	mir-29c	253.99	35.20	877.46	78.36	28.72	1.93
TGGCTCAGTTCAGCAGGAACAG	mir-24-1//mir-24-2	103.54	12.28	816.76	56.93	12.59	0.64
CTGACCTATGAATTGACAGCC	mir-192	481.75	56.54	799.97	70.58	60.15	3.78
AACCCGTAGATCCGATCTTGTG	mir-99a	363.65	21.22	787.65	150.04	48.46	6.15
TCCCTGAGGAGCCCTTTGAGCCTGA	mir-351	18.71	4.40	783.07	92.76	2.59	0.85
AAAAGCTGGGTTGAGAGGGCGA	mir-320	172.30	38.90	677.89	94.17	24.86	2.03
TGAGGTAGGAGGTTGTATAGTT	let-7e	283.91	8.70	656.46	9.92	43.31	1.95
AAACCGTTACCATTACTGAGTT	mir-451	148.59	27.94	621.32	56.02	23.52	2.19
AGCAGCATTGTACAGGGCTATCA	mir-107	41.46	2.94	614.82	35.89	6.78	0.62
AGCTCGGTCTGAGGCCCCTCAGT	mir-423	370.86	36.77	611.15	40.20	60.53	2.98
CAGCAGCACACTGTGGTTTGTA	mir-497	107.56	20.57	578.99	79.56	18.37	1.85
TAGCAGCACAGAAATATTGGC	mir-195	160.00	30.99	518.35	28.53	30.45	4.22
CTGGCCCTCTCTGCCCTTCCGT	mir-328a	119.40	6.98	505.15	44.18	23.83	1.51
CAACCTGGAGGACTCCATGCTG	mir-490	41.39	4.07	445.41	15.34	9.36	1.14
TTTTGCGATGTGTTCCTAATGT	mir-450a	155.89	18.23	404.44	33.20	38.40	2.61
TACAGTACTGTGATAGCTGAA	mir-101b	20.23	1.32	341.49	46.87	6.07	0.52
AACATTCATTGTTGTCGGTGGGT	mir-181d	128.79	28.54	333.10	50.03	37.87	2.67
CAAAGTGCTGTTCGTGCAGGTAG	mir-93	81.84	9.82	318.64	31.97	25.62	0.98
AAGGAGCTCACAGTCTATTGAG	mir-28	90.86	2.54	306.10	14.49	29.88	2.15
TGAGGTAGTAAGTTGTATTGTT	mir-98	164.26	10.31	280.33	4.72	58.62	3.69
CATAAAGTAGAAAGCACTACT	mir-142	110.52	27.55	247.35	31.11	43.65	6.39
ATAAGACGAGCAAAAAGC	mir-208	12.66	3.34	246.53	69.37	5.54	1.43
TCACAGTGAACCGGTCTCTTT	mir-128-1//mir-128-2	105.02	23.05	242.27	39.86	42.65	3.15
TCGGATCCGTCTGAGCTTGGCT	mir-127	91.42	2.36	232.68	4.33	39.28	0.33
CCCAGTGTTCAGACTACCTGTTC	mir-199a	21.15	1.49	229.39	4.53	9.21	0.49
AATGGCGCCACTAGGGTTGTG	mir-652	11.09	1.61	207.91	35.44	5.38	0.13
TGAGAACTGAATTCCATAGGCTGT	mir-146b	117.19	4.90	203.58	12.03	57.74	1.97
TTCCCTTTGTCATCCTATGCCT	mir-204	100.18	27.39	162.06	39.78	61.47	4.10
TAGCACCATTTGAAATCAGTGTT	mir-29b-2//mir-29b-1	78.50	12.53	148.55	14.48	53.01	6.55
CTGTGCGTGTGACAGCGGCTGA	mir-210	50.00	7.35	147.18	3.31	34.11	5.28
CAGTGCAATAGTATTGTCAAAGC	mir-301a	133.50	38.58	143.35	41.88	93.41	1.49

Table S3A Cont.

Mature Sequence	miRNA name	Exact Mature Normalised (RPMM) Mean	SEM	Grouped on Mature Normalised (RPMM) Mean	SEM	Exact/grouped on Mature % Mean	SEM
AGCTACATTGTCTGCTGGGTTTC	mir-221	26.57	8.40	141.30	13.18	17.97	4.69
TCTACAGTGCACGTGTCTCCAG	mir-139	17.17	2.27	139.69	41.11	13.41	2.12
ACAGCAGGCACAGACAGGCAG	mir-214	11.76	6.49	137.25	22.13	7.58	3.10
TAGCAGCACATCATGGTTTACA	mir-15b	96.64	29.23	131.77	35.05	72.28	3.38
TTATAAAGCAATGAGACTGATT	mir-340	84.69	12.81	130.26	13.25	64.30	3.82
GCCCCTGGGCCTATCCTAGAA	mir-331	20.04	1.43	123.79	26.92	17.94	4.03
ACGCCCTTCCCCCCCTTCTTCA	mir-1249	71.65	19.26	119.23	31.46	59.79	1.13
CATGCCTTGAGTGTAGGACTGT	mir-532	62.85	6.87	107.15	8.27	58.47	2.71
TTTGAACCATCACTCGACTCCT	mir-434	95.69	17.45	106.27	20.02	90.34	0.99
TCTTTGGTTATCTAGCTGTATGA	mir-9-1//mir-9-3//mir-9-2	37.94	14.23	100.25	25.81	35.88	4.08
TTATCAGAATCTCCAGGGGTAC	mir-361	49.93	6.10	86.34	7.41	57.46	2.54
TACAGTATAGATGATGTACT	mir-144	54.46	18.69	70.95	25.62	78.66	2.77
TGGCAGTGTCTTAGCTGGTTGT	mir-34a	44.25	6.75	68.46	4.27	64.27	7.65
TAATGCCCCTAAAAATCCTTAT	mir-365	39.70	8.96	66.75	15.26	59.65	0.74
AAGGTTACTTGTTAGTTCAGG	mir-872	23.71	3.12	66.20	7.39	35.65	1.36
TTGTGCTTGATCTAACCATGT	mir-218a-2//mir-218a-1	36.12	10.30	63.76	18.27	57.68	4.26
CAAGTCACTAGTGGTTCCGTTT	mir-224	31.85	5.09	58.13	10.44	55.26	1.39
TGGAGAGAAAGGCAGTTCCTGA	mir-185	28.94	0.39	54.34	3.36	53.70	3.70
AGGCAGTGTAGTTAGCTGATTGC	mir-34c	38.03	1.86	48.83	0.52	77.83	3.05
TAAAGTGCTGACAGTGCAGAT	mir-106b	26.48	3.41	42.28	5.06	62.76	5.15
TGTAACAGCAACTCCATGTGGA	mir-194-1//mir-194-2	29.77	4.47	39.86	2.03	74.36	9.98
TCCGGTTCTCAGGGCTCCACC	mir-671	17.95	0.53	38.82	3.17	46.96	4.43
CCTCTGGGCCCTTCCTCCAGT	mir-326	15.27	2.98	35.66	3.46	42.27	4.85
CAAAGTGCTTACAGTGCAGGTAG	mir-17-1//mir-17-2	10.89	4.53	32.20	5.03	31.54	8.44
AATATAACACAGATGGCCTGT	mir-410	24.75	5.92	26.59	6.73	94.29	2.97
AACTGGCCTACAAAGTCCCAGT	mir-193	19.87	6.85	22.56	7.11	85.43	5.23
TAACAGTCTACAGCCATGGTCG	mir-132	10.77	1.87	22.16	3.81	50.03	8.87

Table S3B

Mature Sequence	miRNA name	Exact Mature Normalised (RPMM) Mean	SEM	Grouped on Mature Normalised (RPMM) Mean	SEM	Exact/grouped on Mature % Mean	SEM
CCCTGTAGAACCGAATTTGTGT	mir-10b	0.00	0.00	1122.49	193.89	0.00	0.00
AGACCCTGGTCTGCACTCTGTC	mir-504	0.32	0.32	252.41	11.73	0.14	0.14
TATTCATTTACTCCCCAGCCTA	mir-664-1//mir-664-2	9.47	0.59	210.25	13.06	4.52	0.29
TTCACAAGAAGGTGTCTTTCAT	mir-3585	2.94	1.56	181.63	20.72	1.45	0.76
TCAGTGCATGACAGAACTTGG	mir-152	6.39	3.25	157.69	10.23	4.32	2.27
AATGACACGATCACTCCCGTTGA	mir-425	8.61	4.50	122.60	10.98	7.74	4.09
CACTCAGTAAGGCATTGTTC	mir-201	7.09	0.28	103.77	14.75	7.14	1.07
TCCAGCATCAGTGATTTTGTTGA	mir-338	1.08	0.56	95.71	7.37	1.04	0.55
TGTGCAAATCCATGCAAAACTGA	mir-19b-1//mir-19b-2	9.31	2.47	72.75	11.77	13.07	2.82
GTCAACACTTGCTGGTTTCC	mir-505	0.76	0.39	71.61	10.05	1.01	0.51
AGCTACATCTGGCTACTGGGT	mir-222	2.25	0.66	66.70	11.40	3.69	1.27
TCTGTCCCTCTTGGCCCTTAG	mir-3577	4.08	0.36	53.56	10.51	8.43	2.15
TGATATGTTTGATATATTAGGT	mir-190	3.99	2.03	53.40	11.04	6.26	3.26
ATATAATACAACCTGCTAAGTG	mir-374	6.53	1.34	51.88	7.73	12.45	1.54
TGTCAGTTTGTCAAATACCCC	mir-223	6.46	3.38	51.09	5.43	13.72	6.90
TGCTGACCCCTAGTCCAGTGC	mir-345	3.42	2.23	43.42	5.36	7.60	4.28
TCCCTGTCCTCCAGGAGCTCACG	mir-339	5.83	1.02	43.13	3.15	13.39	1.89
GTGAAATGTTTAGGACCACTAG	mir-203	6.31	2.30	35.29	7.22	16.71	3.23
TTTGGCAATGGTAGAACTCACACCG	mir-182	6.42	2.76	32.64	4.01	18.98	6.67
TGGACGGAGAACTGATAAGGGT	mir-184	2.69	0.47	29.54	21.32	31.79	18.49
TAGTAGACCGTATAGCGTACG	mir-411	9.15	0.86	22.10	1.60	41.65	4.19
TAAAGTGCTTATAGTGCAGGTAG	mir-20a	9.25	1.26	19.51	4.53	49.44	5.30
TCAGGCTCAGTCCCCTCCCGAT	mir-484	4.88	1.97	17.99	5.68	24.86	7.90
TTGAAAGGCTGTTTCTTGGTC	mir-488	4.18	1.43	16.68	4.40	32.40	17.35
TAATACTGCCGGGTAATGATG	mir-200c	0.00	0.00	15.81	4.11	0.00	0.00
CAGTGGTTTTACCCTATGGTAG	mir-140	6.87	1.85	13.24	0.50	51.15	11.93
TAACACTGTCTGGTAAAGATGG	mir-141	0.32	0.32	13.14	3.95	1.52	1.52
TATACAAGGGCAAGCTCT	mir-381	0.00	0.00	12.89	1.68	0.00	0.00
GGCAGAGGAGGGCTGTTCTTCCC	mir-298	7.48	2.15	12.83	3.64	57.94	4.83
CTCGGGGATCATCATGTCACGA	mir-542	1.81	1.02	11.85	5.00	11.62	5.82
GAAGTTCTGTTATACACTCAGG	mir-148b	0.00	0.00	11.15	1.77	0.00	0.00
TGTCTGCCTGAGTGCCTGCCTCT	mir-346	6.56	2.05	10.93	1.82	64.44	21.20
AATTGCACGGTATCCATCTGT	mir-363	6.02	0.52	10.74	0.89	56.65	5.93
GCACTGAGATGGGAGTGGTGTA	mir-674	0.32	0.32	9.44	1.95	2.38	2.38
AAGGGATTCTGATGTTGGTCACACT	mir-541	2.91	1.47	8.32	2.50	27.78	14.70
AGGCAAGATGCTGGCATAGCTG	mir-31	5.22	2.05	8.07	2.35	75.00	25.00
AGCTGGTGTTGTGAATCAGGCCG	mir-138-2//mir-138-1	5.57	3.47	7.84	4.60	68.75	6.25
TATTGCACTCGTCCCGGCCTCC	mir-92b	1.39	0.82	7.00	1.79	15.56	8.68
TGTAAACAATTCCTAGGCAATGT	mir-384	4.18	2.47	5.89	3.58	72.12	2.88

Table S3 MiRNA reads annotated to previously characterised rat sequences (by alignment to miRBase V18) normalised to the total number of annotated sequences (reads per million mapped (RPMM)) The figure in the column labelled 'Exact Mature' refers to the number of reads matching the canonical mature miRNA sequence in miRBase, while 'Grouped on Mature' refers to all reads matching the mature sequence but allowing for up to 2 mismatches within the sequence and/or 3 additions/ deletions from either the 5' or 3' ends. A: 106 miRNAs with ≥10 reads of the 'exact mature' sequence in any one sample prior to normalisation. B: An additional 39 miRNAs with <10 'exact mature' reads but ≥10 'grouped on mature' reads in any one sample prior to normalisation.
