# Supplementary material for: Distinctive Profile of IsomiR Expression and Novel MicroRNAs in Rat Heart Left Ventricle
Source: PLoS One. 2013 Jun 14;8(6):e65809. doi: 10.1371/journal.pone.0065809 (PMC3683050; doi:10.1371/journal.pone.0065809)
Supplement: Table S4 — MiRNA annotated to sequences not previously characterised in rat but identified in other species. (PDF) [file pone.0065809.s006.pdf]

Table S4A

Mature Sequence	miRNA name	Exact Mature Normalised (RPMM) Mean	SEM	Grouped on Mature Normalised (RPMM) Mean	SEM	Exact/grouped on Mature % Mean	SEM
TCCTGTACTGAGCTGCCCCGAG	hsa-mir-486//mir-3107	48568.44	6048.85	117242.03	12999.51	41.30	0.75
TCTGGCTCCGTGTCTTCACTCCC	hsa-mir-149	946.12	150.45	2930.49	308.21	31.91	1.78
TAGCAGCACATAATGGTTTGT	bta-mir-15a	319.73	19.67	338.36	23.18	94.61	1.26
TGCGGGGCTAGGGCTAACAGCA	hsa-mir-744	52.69	4.03	259.03	33.62	20.68	1.31
CACGCTCATGCACACACCCACA	cfa-mir-574	96.25	11.34	181.70	9.65	52.71	4.25
AAGGTAGATAGAACAGGTCTTG	cfa-mir-1839	33.09	4.28	158.62	13.57	21.36	4.01
CTCCTGGCTGGCTCGCCA	oan-mir-1386	62.06	11.12	155.09	13.55	39.75	5.74
TAGCAGCACATAATGGTTTGTG	age-mir-15a	40.54	4.42	152.80	13.04	27.50	5.70
TATGGAGGTCTCTGTCTGACT	mmu-mir-1843	44.60	10.34	106.20	22.18	41.74	1.58
AACTGGCCCACAAAGTCCCGCT	eca-mir-193b	61.70	27.35	92.79	38.14	64.79	3.33
TCTCGCTGGGGCCTCCA	hsa-mir-720	32.85	6.48	52.16	5.11	61.76	7.88
ATGGAGGTCTCTGTCTGACTT	mmu-mir-1843b	14.91	2.72	32.00	5.96	46.68	2.63
GGGGCTGGGCGCGCGCC	hsa-mir-4492	20.83	10.24	29.76	14.60	76.52	12.39
ATCTCGCTGGGGCCTCCA	mmu-mir-720	20.55	1.54	22.83	2.61	91.03	4.68
CTCACCTGGAGCATGTTTTCT	mmu-mir-1983	11.52	4.20	19.50	4.87	56.06	6.06

Table S4B

Mature Sequence	miRNA name	Exact Mature Normalised (RPMM) Mean	SEM	Grouped on Mature Normalised (RPMM) Mean	SEM	Exact/grouped on Mature % Mean	SEM
CTGGACGCGAGCTGGGCCC	mmu-mir-5115	0.44	0.44	4283.42	1853.07	0.01	0.01
TGAGGTAGTAGTTTGTACAGTT	bta-let-7g	0.00	0.00	458.94	40.94	0.00	0.00
TCAGTGCACTACAGAACTTTGT	bta-mir-148a//mir-148	0.00	0.00	306.39	29.74	0.00	0.00
ATCCCACCTCTGCCACCA	hsa-mir-1260a	0.00	0.00	291.33	17.15	0.00	0.00
TATTGCACTTGTCCCGGCCTGT	dre-mir-92a-1//mir-92a-2//mir-92-2	0.00	0.00	256.67	31.15	0.00	0.00
GTATCCCACTTCTGACACCA	bta-mir-2478	0.00	0.00	225.61	42.77	0.00	0.00
TTCAAGTAATCCAGGATAGGCT	dre-mir-26a-1//mir-26a-2//mir-26a-3/ /mir-26//mir-26-1//mir-26-2	0.00	0.00	135.04	20.98	0.00	0.00
AAAGTTCTGAGACACTCCGACT	hsa-mir-148a	0.00	0.00	134.97	18.60	0.00	0.00
TGTTGCGGACCAGGGGAATCCGA	mmu-mir-5109	0.00	0.00	116.37	13.50	0.00	0.00
TTGGTCCCCTTCAACCAGCTGT	xla-mir-133a//mir-133	0.00	0.00	95.41	24.89	0.00	0.00
TCGTACCGTGAGTAATAATGC	dre-mir-126a//mir-126	0.00	0.00	90.46	12.25	0.00	0.00
TTGGTCCCCGTCAACCAGCTGT	sme-mir-133a	0.00	0.00	70.62	19.55	0.00	0.00
TGAGGTAGTAGTTTGTACAGT	gga-let-7g	0.00	0.00	53.01	3.71	0.00	0.00
ATATCCGGCTCGAAGGACCA	bmo-mir-2779	0.00	0.00	48.29	1.61	0.00	0.00
TGGAATGTAAAGAAGTATGTAC	pma-mir-1c	0.00	0.00	41.79	11.73	0.00	0.00
TTCACAGTGGCTAAGTTCTGCA	dre-mir-27b	0.00	0.00	36.26	1.33	0.00	0.00
ATCCCACCACTGCCACCAT	hsa-mir-1260b	0.00	0.00	34.48	2.06	0.00	0.00
ACCCGTCCCGTTCGTCCCCGGA	hsa-mir-1247	1.77	1.77	33.20	9.99	3.33	3.33
GGATCCGAGTCACGGCACCA	hsa-mir-4454	0.00	0.00	31.61	4.55	0.00	0.00
TTCAAGTAATCCAGGATAGGTT	dre-mir-26b	0.00	0.00	31.39	3.67	0.00	0.00
ACTGGACTTGGAGTCAGAAGGC	bta-mir-378-2//mir-378-1	0.00	0.00	30.90	4.94	0.00	0.00
ACTGGACTTGGAGTCAGAAA	hsa-mir-378d-2//mir-378d-1	0.00	0.00	28.28	4.19	0.00	0.00
AACTGGCCCACAAAGTCCCGCTTT	gga-mir-193b	6.96	2.90	26.77	6.07	27.04	13.16
TTAATGCTAATTGTGATAGGGGT	mmu-mir-155	4.21	0.56	25.43	1.25	16.44	1.39
CGAATCCCACTCCAGACACCA	mmu-mir-3968	0.00	0.00	24.28	6.79	0.00	0.00
TGAGTGTGTGTGTGTGAGTGTGT	hsa-mir-574	2.47	1.37	19.25	2.81	11.11	5.88
TCCCACCGCTGCCACCC	hsa-mir-1280	0.00	0.00	18.61	6.06	0.00	0.00
GGGCTGGAGAGATGGCTCAG	mmu-mir-3473b	0.63	0.63	18.18	3.81	2.78	2.78
CCTATTCTTGATTACTTGTTT	sha-mir-26a	0.44	0.44	17.98	4.29	1.67	1.67
ACTGGACTTGGAGTCAGAAGAGTGG	hsa-mir-378c	0.00	0.00	17.79	2.07	0.00	0.00
GCGGGCGGGGCCGGGGGCGGGG	mmu-mir-5126	0.00	0.00	17.04	9.54	0.00	0.00
TTCACAGTGGCTAAGTTCCGC	xtr-mir-27a	0.00	0.00	15.99	1.37	0.00	0.00
TGAGGTAGTAGGTTGTATAGT	bmo-let-7	0.00	0.00	13.74	2.90	0.00	0.00
AAAGGCTAGGCTCACAACCAAA	mmu-mir-690	2.79	2.16	12.54	1.63	21.43	14.87
CAACGGAATCCCAAAAGCAGCTG	aca-mir-191	0.00	0.00	11.91	3.15	0.00	0.00
CAAAGAATTCTCCTTTTGGGCTT	ssc-mir-186	0.00	0.00	11.84	2.46	0.00	0.00
TGAGGTAGTAGGTTGTATAG	tca-let-7	0.00	0.00	11.72	1.18	0.00	0.00
GCTTCTGGCGCCAAGCTTCCGTCC	mmu-mir-5102	0.00	0.00	11.49	4.47	0.00	0.00
TCACTCTGTAGACCAGGCTGG	mmu-mir-3470b	0.00	0.00	10.89	7.28	0.00	0.00
TGAGATGAAGCACTGTAGCTC	aca-mir-143	0.00	0.00	10.86	3.61	0.00	0.00
TGGTCCCCTTCAACCAGCTGTA	cin-mir-133	0.00	0.00	10.39	2.15	0.00	0.00
GTTCATGTCCCTGTTCAGGCGCCA	mmu-mir-5097	0.00	0.00	10.16	3.38	0.00	0.00
GGATATGATGACTGATTATCTGAAA	gga-mir-3535	0.00	0.00	9.91	3.26	0.00	0.00
TTGGTCCCCTTCAACCAGCTG	bfl-mir-133	0.00	0.00	9.81	1.45	0.00	0.00
TGGAATGTAAAGAAGTATGTAG	cte-mir-1	0.00	0.00	8.51	2.51	0.00	0.00
TGTAAACATCCTACACTCTCAGCT	gga-mir-30c-1//mir-30c	0.00	0.00	7.88	2.20	0.00	0.00
GCGGTGGGCCTGGAAGCCT	sha-mir-716a	0.00	0.00	7.73	0.89	0.00	0.00
TCCCTGTGGTCTAGTGGTTAG	ssc-mir-2476-1//mir-2476-2//mir-2476-3	1.81	1.02	7.69	3.32	37.78	31.35
TGGAATGTAAAGAAGTATGTA	cbr-mir-1	0.00	0.00	7.38	3.01	0.00	0.00
TGAGATGAAGCACTGTAGCTCG	bta-mir-143	0.00	0.00	7.19	2.29	0.00	0.00
CTTGGCACCTAGTAAGCACT	eca-mir-1271	0.00	0.00	5.94	5.02	0.00	0.00
TGGAATGTAAAGAAGTATGTAT	hsa-mir-1-1//mir-1-2//mir-1//mir-1a-1//mir-1a-2	0.00	0.00	5.86	2.78	0.00	0.00
TAACGGCCGCGGTACCCTAA	hsa-mir-4485	0.00	0.00	4.68	3.44	0.00	0.00

Table S4 MiRNA annotated to sequences not previously characterised in rat but identified in other species (novel orthologs; aligned to miRBase V18) normalised to total annotated sequences (reads per million mapped (RPMM))for **A** 15 miRNAs with ≥10 reads of the 'exact mature' sequence in any one sample prior to normalisation and **B** an additional 53 miRNAs with ≥10 'grouped on mature' reads (allowing for up to 2 mismatches within the sequence and/or 3 additions/deletions from either the 5' or 3' ends) in any one sample prior to normalisation.
