# Supplementary material for: Distinctive Profile of IsomiR Expression and Novel MicroRNAs in Rat Heart Left Ventricle
Source: PLoS One. 2013 Jun 14;8(6):e65809. doi: 10.1371/journal.pone.0065809 (PMC3683050; doi:10.1371/journal.pone.0065809)
Supplement: Table S5 — *strand miRNA annotated to previously characterised sequences. (PDF) [file pone.0065809.s007.pdf]

Table S5A

Mature* Sequence	miRNA name	Exact Mature Normalised (RPMM)		Grouped on Mature Normalised (RPMM)		Exact/grouped on Mature %		Mature/* Fold difference
		Mean	SEM	Mean	SEM	Mean	SEM	
CATTATTACTTTTGGTACGCG	rno-mir-126*	6229.70	1046.59	7990.04	1136.06	77.40	2.44	1.3210
CTAGACTGAGGCTCCTTGAGG	rno-mir-151*	1242.77	182.20	5568.43	835.89	22.36	0.24	3.3165
CACTAGATTGTGAGCTCCTGGA	rno-mir-28*	1031.21	105.96	2367.74	188.68	43.40	0.98	7.7353
CTTTCAGTCGGATGTTTACAGC	rno-mir-30e*	912.62	30.24	1505.23	21.87	60.62	1.60	0.0960
CTATACGACCTGCTGCCTTTCT	rno-let-7d*	883.81	98.43	1215.29	136.35	72.74	0.14	0.6198
TGAGGGGCAGAGAGCGAGACTTTT	rno-mir-423*	98.81	9.69	753.84	74.42	13.17	0.80	1.2335
CTTTCAGTCGGATGTTTGCAGC	rno-mir-30a*	303.61	15.47	720.18	46.27	42.25	1.03	0.0164
ACAAGTCAGGCTCTTGGGACCT	rno-mir-125b-2*	176.53	15.64	559.00	32.26	31.52	1.67	0.1264
AGCTGGTAAAATGGAACCAAAT	rno-mir-133a*	176.14	15.10	265.87	31.38	66.77	2.20	0.0025
GTGCCTACTGAGCTGAAACAGT	rno-mir-24-2*	107.49	11.98	228.65	24.41	46.97	0.24	0.2921 #
AGTTCTTCAGTGGCAAGCTTTA	rno-mir-22*	62.17	6.44	184.99	17.51	33.71	2.08	0.0012
CTGGGAGAAGGCTGTTTACTCT	rno-mir-30c-2*	119.97	14.81	184.73	15.68	64.51	3.02	0.0146 #
CTCCTGACTCCAGGTCCTGTGT	rno-mir-378*	119.34	15.22	170.93	19.00	69.57	1.56	0.0071
ACCATCGACCGTTGAGTGGACC	rno-mir-181c*	45.89	14.14	141.09	24.71	30.69	5.39	0.0847
CCCCCAGGTGTGATTCTGATTCGT	rno-mir-361*	21.22	0.76	138.23	14.47	15.63	1.38	1.6010
ACCATCGACCGTTGATTGTACC	rno-mir-181a-1*	61.91	3.53	105.11	5.76	58.93	1.72	0.0090 #
GAGCTTTTGGCCCGGGTTATAC	rno-mir-208*	67.37	21.34	103.98	24.71	62.01	5.93	0.4218
TGAACTATTGCAGTAGCCTCCT	rno-mir-872*	75.09	4.44	85.79	2.40	87.38	2.92	1.2959
CTATACAATCTACTGTCTTTCC	rno-let-7a-1//let-7c-2*	8.64	2.60	84.98	7.30	9.99	2.75	0.0081
CTGGGAGAGGGTTGTTTACTCC	rno-mir-30c-1*	44.85	9.58	80.77	12.73	54.60	3.10	0.0146 #
GCAGTCCACGGGCATATACACT	rno-mir-455*	16.21	2.53	57.98	3.98	27.76	2.91	4.7412
GGATATCATCATATACTGTAAGT	rno-mir-144*	43.54	15.99	57.09	18.65	73.54	4.70	0.8047
CTTTCAGTCAGATGTTTGCTGC	rno-mir-30d*	18.49	3.42	50.42	10.11	37.60	4.07	0.0020
CCAATATTGGCTGTGCTGCTCCA	rno-mir-195*	8.04	1.21	35.80	5.75	23.52	4.21	0.0691
AACCTGTTGAACAACTGAACCC	rno-mir-582*	11.12	2.76	30.87	5.94	35.65	4.89	6.2961
AGGTTCTGTGATACACTCCGACT	rno-mir-152*	16.91	2.54	19.63	2.26	85.91	7.18	0.1245
AGCCACTGCCCACAGCACACTG	rno-mir-210*	9.75	2.43	13.17	1.91	73.89	12.41	0.0895
CATCATCGTCTCAAATGAGTCT	rno-mir-136*	8.49	1.02	9.44	1.95	92.86	7.14	4.9839
AGGGCCCCCCCTCAATCCTGT	rno-mir-296*	4.24	3.60	5.00	3.75	71.15	21.15	6.4438

Table S5B

Mature* Sequence	miRNA name	Exact Mature Normalised (RPMM)		Grouped on Mature Normalised (RPMM)		Exact/grouped on Mature %		Mature/* Fold difference
		Mean	SEM	Mean	SEM	Mean	SEM	
TACCACAGGGTAGAACCACGG	rno-mir-140*	6.97	0.94	1317.90	143.75	0.55	0.11	99.5617
GGATTCCTGGAAATACTGTTC	rno-mir-145*	0.00	0.00	396.04	165.44	0.00	0.00	0.2717
AAACATGAAGCGCTGCAACA	rno-mir-322*	7.95	1.31	316.02	13.03	2.49	0.30	0.3598
CTATACAACCTACTGCCTTCCC	rno-let-7b*	0.32	0.32	236.06	24.74	0.13	0.13	0.0973
ACCACCAACCGTTGACTGT	rno-mir-181a-2*	4.15	1.84	118.15	13.34	3.27	1.14	0.0090 #
CAAGCTCGTTTCTATGGGTCTG	rno-mir-99a*	5.48	2.61	81.58	5.15	6.44	2.69	0.1036
AGAGCTTAGCTGATTGGTGAACAG	rno-mir-27b*	0.44	0.44	80.99	11.87	0.44	0.44	0.0032
CCGCACTGTGGGTACTTGCTGC	rno-mir-106b*	2.35	2.35	67.59	8.47	2.78	2.78	1.5988
GGAGTATTGTTTCCGCTGCCTGG	rno-mir-503*	3.10	3.10	44.55	17.31	3.95	3.95	23.6585
ATCAACAGACATTAATTGGG	rno-mir-421*	0.00	0.00	38.23	7.30	0.00	0.00	32.8394
CTATACAATCTATTGCCTTCC	rno-let-7f-1*	1.71	1.11	30.31	4.89	5.42	2.92	0.0023
CAAGCTCGTGTCTGTGGGTCCG	rno-mir-99b*	1.49	1.05	24.64	5.60	5.29	2.90	0.0056
TCACTTCAGGATGTACCACCCA	rno-mir-547*	2.85	1.97	23.30	2.29	10.97	6.94	3.2432
GGTGCAGTGCTGCATCTCTGG	rno-mir-143*	1.96	1.15	22.64	3.94	8.75	5.91	0.0002
CTGCGCAAGCTACTGCCTTGCT	rno-let-7i*	9.47	0.59	21.59	2.74	45.00	5.00	0.0039
CCTGTTCTCCATTACTTGGCTC	rno-mir-26b*	9.38	2.70	20.02	4.35	45.24	4.76	0.0038
TGACCGATTTCTCCTGGTGTTC	rno-mir-29c*	0.00	0.00	18.24	1.92	0.00	0.00	0.0208
CTATACGGCCTCCTAGCTTTCC	rno-let-7e*	7.19	1.69	18.15	3.25	38.91	2.00	0.0276
CTGGCTGGGGAAAATGATTGG	rno-mir-664-2*	0.76	0.39	17.13	1.01	4.46	2.25	0.0815
CTATACAGTCTACTGTCTTTC	rno-let-7f-2*	0.44	0.44	16.09	2.42	3.33	3.33	0.0023 #
AACATCACAGCAAGTCTGTGCT	rno-mir-499*	2.25	0.66	14.89	2.72	15.67	4.74	0.0120
ACAACAAATCACAGTCTGCCAT	rno-mir-7a-1*	0.00	0.00	14.76	0.59	0.00	0.00	4.0919
TCAGTTATCACAGTGCTGATGC	rno-mir-101a*	0.00	0.00	13.06	4.26	0.00	0.00	0.0026
CTATACAACTTACTACTTTCC	rno-mir-98*	0.32	0.32	11.37	2.44	4.17	4.17	0.0405
GCTGCACTTGGATTTCGTTCCC	rno-mir-191*	0.76	0.39	11.28	1.44	7.79	4.17	0.0007
GTGCCTACTGAGCTGATATCAG	rno-mir-24-1*	1.84	0.92	9.94	1.81	16.67	8.82	0.2921 #
ACTGATTTCTTTTGGTGTTCAG	rno-mir-29a*	1.84	0.92	9.05	1.06	18.33	9.28	0.0011
CAACAGCAGTCGATGGGCTGTC	rno-mir-21*	0.89	0.89	8.51	2.51	8.33	8.33	0.0031
AACAATATCCTGGTGCTGAGTG	rno-mir-338*	0.44	0.44	7.73	0.89	6.67	6.67	0.0807
AATGCACCCGGGCAAGGATTTGG	rno-mir-501*	0.00	0.00	6.26	4.91	0.00	0.00	∞

Table S5 * strand miRNA annotated to previously characterised sequences (aligned to miRBase V18) normalised to total annotated sequences (reads per million mapped (RPMM)) for **A** 28 miRNAs with ≥10 reads of the exact mature* sequence in any one sample prior to normalisation and **B** an additional 30 miRNAs with ≥10 reads grouped on mature* sequence (allowing for up to 2 mismatches within the sequence and/or 3 additions/deletions from either the 5’ or 3’ ends) in any one sample prior to normalisation. # indicates fold differences calculated by combining reads for samples with identical mature sequences. Note absence of any miR-501 mature reads.
