# Supplementary material for: Distinctive Profile of IsomiR Expression and Novel MicroRNAs in Rat Heart Left Ventricle
Source: PLoS One. 2013 Jun 14;8(6):e65809. doi: 10.1371/journal.pone.0065809 (PMC3683050; doi:10.1371/journal.pone.0065809)
Supplement: Table S6 — Shortlist of genes relevant to cardiac contractile function predicted to be targeted by the top 16 highest detected miRNAs. (PDF) [file pone.0065809.s008.pdf]

Table S6

Gene Name	Protein	Function in heart
ABCC8	ATP-binding cassette, sub-family C (CFTR/MRP), member 8 (SUR1)	Ion Homeostasis/contraction (I_{KATP})
ABCC9	ATP-binding cassette, sub-family C (CFTR/MRP), member 9 (SUR2)	Ion Homeostasis/contraction (I_{KATP})
ACTC1	Actin, alpha, cardiac muscle	Contraction
ACTG1	Actin, gamma 1	Cytoskeleton
ACTN1	Actinin, alpha 1	Cytoskeleton
ACTN2	Actinin alpha 2	Cytoskeleton
ACTN4	Actinin alpha 4	Cytoskeleton
ADCY5	Adenylate cyclase 5	Ca^{2+} signalling
ADCY6	Adenylate cyclase 6	Ca^{2+} signalling
ADCY7	Adenylate cyclase 7	Ca^{2+} signalling
ADORA2A	Adenosine receptor 2A	Ca^{2+} signalling
ADRA1B	Adrenergic receptor, alpha-1B	Ca^{2+} signalling
ADRA1D	Adrenergic receptor, alpha-1D	Ca^{2+} signalling
ADRB1	Adrenergic receptor, beta-1	Ca^{2+} signalling
ADRB2	Adrenergic receptor, beta-2	Ca^{2+} signalling
ATP1B1	ATPase, Na^+/K^+ transporting, beta 1 polypeptide	Ion Homeostasis/contraction
ATP2A2	ATPase, Ca^{2+} transporting, cardiac muscle, slow twitch 2 (SERCA2)	Contraction/Ca^{2+} Homeostasis
ATP2A3	ATPase, Ca^{2+} transporting, ubiquitous (SERCA3)	Contraction/Ca^{2+} Homeostasis
ATP2B1	ATPase, Ca^{2+} transporting, plasma membrane 1 (PMCA1)	Ca^{2+} Homeostasis/contraction
ATP2B2	ATPase, Ca^{2+} transporting, plasma membrane 2 (PMCA2)	Ca^{2+} Homeostasis/contraction
ATP2B4	ATPase, Ca^{2+} transporting, plasma membrane 4 (PMCA4)	Ca^{2+} Homeostasis/contraction
CACNA2D1	Calcium channel, voltage-dependent, alpha2/delta subunit 1	Contraction ($I_{Ca,L}$)
CACNA2D2	Calcium channel, voltage-dependent, alpha2/delta subunit 2	Contraction ($I_{Ca,L}$)
CACNB2	Calcium channel, voltage-dependent, beta 2 subunit	Contraction ($I_{Ca,L}$)
CACNB3	Calcium channel, voltage-dependent, beta 3 subunit	Contraction ($I_{Ca,L}$)
CACNG4	Calcium channel, voltage-dependent, gamma 3 subunit	Contraction ($I_{Ca,L}$)
CALM1	Calmodulin 1	Ca^{2+} signalling, gene expression
CALM3	Calmodulin 3	Ca^{2+} signalling, gene expression
CAMK2D	Calcium/calmodulin-dependent protein kinase II delta	Gene expression
CAMK2G	Calcium/calmodulin-dependent protein kinase II gamma	Gene expression
CAMK2N1	Calcium/calmodulin-dependent protein kinase II inhibitor 1	Gene expression
CAMKK1	Calcium/calmodulin-dependent protein kinase kinase 1, alpha	Gene expression
CAMKK2	Calcium/calmodulin-dependent protein kinase kinase 2, beta	Gene expression
CAPZA2	Capping protein (actin filament) muscle Z-line, alpha 2	Cytoskeleton
CAPZB	Capping protein (actin filament) muscle Z-line, beta	Cytoskeleton
CASQ2	Calsequestrin 2 (cardiac muscle)	Ca^{2+} homestasis
CDH2	Cadherin-2	Cytoskeleton
COX5A	Cytochrome c oxidase subunit 5A	Mitrochondial ion homeostasis
DAG1	Dystroglycan 1	Cytoskeleton
DMD	Dystrophin	Cytoskeleton
EDNRA	Endothelin receptor type A	Ca^{2+} signalling
EDNRB	Endothelin receptor type B	Ca^{2+} signalling
EGFR	Epidermal growth factor receptor 1	Ca^{2+} signalling
ERBB3	Epidermal growth factor receptor B-3	Ca^{2+} signalling
F2R	Proteinase-activated receptor 1 (PAR1)	Ca^{2+} signalling
FOXE1	Forkhead box E1 (thyroid transcription factor 2)	Gene expression
FOXO1	Forkhead box O1	Gene expression
FOXP1	Forkhead box P1	Gene expression
GJA1	Gap junction protein, alpha 1	Cell communication
GJA5	Gap junction protein, alpha 5	Cell communication
GNAI1	G protein, alpha inhibiting activity polypeptide 1	Ca^{2+} signalling
GNAI2	G protein, alpha inhibiting activity polypeptide 2	Ca^{2+} signalling
GNAI3	G protein, alpha inhibiting activity polypeptide 3	Ca^{2+} signalling
GNAO1	G protein, alpha activating activity polypeptide O	Ca^{2+} signalling
GRIN2A	Glutamate receptor, ionotropic, N-methyl D-aspartate 2D	Ca^{2+} signalling
GRIN2D	Glutamate receptor, ionotropic, N-methyl D-aspartate 2A	Ca^{2+} signalling
HCN1	Hyperpolarization-activated cyclic nucleotide-gated potassium channel 1	Pacemaking (I_f)
HCN4	Hyperpolarization-activated cyclic nucleotide-gated potassium channel 4	Pacemaking (I_f)
IGF1	Insulin-like growth factor 1	Gene expression
IRX5	Iroquois homeobox 5	Gene expression
ITGA1	Integrin alpha 1	Cytoskeleton
ITGA6	Integrin, alpha 6	Cytoskeleton
ITGB1	Integrin beta 1 (fibronectin receptor beta)	Cytoskeleton
ITGB3	Integrin beta 3	Cytoskeleton
ITPK1	Inositol 1,3,4-triphosphate 5/6 kinase	Ca^{2+} signalling
ITPR1	Inositol 1,4,5-triphosphate receptor, type 1	Ca^{2+} signalling
JUP	Junction plakoglobin	Cytoskeleton
KCNA4	Potassium voltage-gated channel, shaker-related subfamily, member 4 (Kv1.4)	Action potential (I_{TO})
KCNAB1	Potassium voltage-gated channel, shaker-related subfamily, beta member 1 (Kvβ1)	Action potential (I_{Kur})
KCNIP2	Kv channel-interacting protein 2 (KChIPs)	Action potential (I_{TO})
KCNJ3	Potassium inwardly-rectifying channel, subfamily J, member 3	Action potential (I_{KACH})
KCNJ11	Potassium inwardly rectifying channel, subfamily J, member 11	Ion Homeostasis/contraction (I_{KATP})
KCNJ12	Potassium inwardly-rectifying channel, subfamily J, member 12	Action potential (I_{K1})
KCNK1	Potassium channel, subfamily K, member 1 (TWIK)	Ion Homeostasis/contraction (I_{KP})
LMNA	Lamin A/C	Cytoskeleton
MYLK	Myosin light chain kinase	Contraction
NFAT5	Nuclear factor of activated T-cells 5, tonicity-responsive	Gene expression
NFATC1	Nuclear factor of activated T-cells, cytoplasmic, calcineurin-dependent 1	Gene expression

Table S6 Cont.

Gene Name	Protein	Function in heart
NFATC2	Nuclear factor of activated T-cells, cytoplasmic, calcineurin-dependent 2	Gene expression
P2RX4	P2X purinoceptor 4	Ca^{2+} signalling
PDGFRA	Platelet-derived growth factor receptor, alpha polypeptide	Gene expression
PDGFRB	Platelet-derived growth factor receptor, beta polypeptide	Gene expression
PIAS3	Protein inhibitor of activated STAT, 3 (kchap)	Ion Homeostasis
PLA1A	Phospholipase A1 member A	Ca^{2+} signalling
PLA2G15	Phospholipase A2, group XV	Ca^{2+} signalling
PLA2G4A	Phospholipase A2, group IVA (cytosolic, calcium-dependent)	Ca^{2+} signalling
PLA2G6	Phospholipase A2, group VI (cytosolic, calcium-independent)	Ca^{2+} signalling
PLCB1	Phospholipase C, beta 1 (phosphoinositide-specific)	Ca^{2+} signalling
PLCB4	Phospholipase C, beta 4	Ca^{2+} signalling
PLCD1	Phospholipase C, delta 1	Ca^{2+} signalling
PLCG1	Phospholipase C, gamma 1	Ca^{2+} signalling
PLCL1	Phospholipase C-like 1	Ca^{2+} signalling
PPP3CA	Protein phosphatase 3, catalytic subunit, alpha (calcineurin)	Gene expression
PRKAA2	Protein kinase, AMP-activated, alpha 2 catalytic subunit	Ca^{2+} signalling
PRKAB2	Protein kinase, AMP-activated, beta 2 non-catalytic subunit	Ca^{2+} signalling
PRKAG2	Protein kinase, AMP-activated, gamma 2 non-catalytic subunit	Ca^{2+} signalling
PRKAR1A	Protein kinase, camp-dependent, regulatory, type I, alpha	Ca^{2+} signalling
PRKAR2B	Protein kinase, camp-dependent, regulatory, type II, beta	Ca^{2+} signalling
PRKCD	Protein kinase C, delta	Ca^{2+} signalling
PRKCE	Protein kinase C, epsilon	Ca^{2+} signalling
PRKCH	Protein kinase C, eta	Ca^{2+} signalling
PRKX	Protein kinase, X-linked	Ca^{2+} signalling
PTGFR	Prostaglandin F receptor	Ca^{2+} signalling
SCN3B	Sodium channel, voltage-gated, type III, beta	Action potential (I_{Na})
SCN5A	Sodium channel, voltage-gated, type V, alpha subunit	Action potential (I_{Na})
SLC8A1	Plasma membrane Na/Ca exchanger, member 1 (NCX1)	Ca^{2+} Homeostasis/Action potential/Contraction(I_{NaCa})
SLC9A1	Plasma membrane Na/H antiporter	Ion Homeostasis
SLC9A6	Mitrochondrial Na/H antiporter	Ion Homeostasis
TBX2	T-box 2	Gene expression
TCF7	Transcription factor 7	Gene expression
TPM3	Tropomyosin 3	Contraction
TRPC1	Transient receptor potential channel, subfamily C, member 1	Ca^{2+} homeostasis (I_{NSCC})
VDAC1	Voltage-dependent anion channel 1	Mitrochondial ion homeostasis
VDAC3	Voltage-dependent anion channel 3	Mitrochondial ion homeostasis

TableS6 Shortlist of genes relevant to cardiac contractile function predicted to be targeted by the top 16 highest detected miRNAs.
